# Autogene cevumeran with or without atezolizumab in advanced solid tumors: a phase 1 trial

**DOI:** 10.1038/s41591-024-03334-7

**Published:** 2025-01-06

**Authors:** Juanita Lopez, Thomas Powles, Fadi Braiteh, Lillian L. Siu, Patricia LoRusso, Claire F. Friedman, Ani S. Balmanoukian, Michael Gordon, Jeffrey Yachnin, Sylvie Rottey, Ioannis Karydis, George A. Fisher, Marcus Schmidt, Martin Schuler, Ryan J. Sullivan, Howard A. Burris, Vladimir Galvao, Brian S. Henick, Luc Dirix, Dirk Jaeger, Patrick A. Ott, Kit Man Wong, Guy Jerusalem, Aglaia Schiza, Lawrence Fong, Neeltje Steeghs, Rom S. Leidner, Achim Rittmeyer, Scott A. Laurie, Eelke Gort, Raid Aljumaily, Ignacio Melero, Rachel L. Sabado, Ina Rhee, Michael R. Mancuso, Lars Muller, Gregg D. Fine, Mahesh Yadav, Leesun Kim, Vincent J. P. Leveque, Alberto Robert, Martine Darwish, Ting Qi, Jiawen Zhu, Jingbin Zhang, Patrick Twomey, Gautham K. Rao, Donald W. Low, Chris Petry, Amy A. Lo, Jill M. Schartner, Lélia Delamarre, Ira Mellman, Martin Löwer, Felicitas Müller, Evelyna Derhovanessian, Andrea Cortini, Luisa Manning, Daniel Maurus, Sebastian Brachtendorf, Verena Lörks, Tana Omokoko, Eva Godehardt, Dirk Becker, Christine Hawner, Christine Wallrapp, Christian Albrecht, Christoph Kröner, Arbel D. Tadmor, Jan Diekmann, Mathias Vormehr, Anette Jork, Anna Paruzynski, Maren Lang, Jonathon Blake, Oliver Hennig, Andreas N. Kuhn, Ugur Sahin, Özlem Türeci, D. Ross Camidge

**Affiliations:** 1 The Royal Marsden Hospital and the Institute of Cancer Research, Sutton, UK; 2 Barts Cancer Institute, Centre for Experimental Cancer Medicine, Queen Mary University of London, London, UK; 3 Comprehensive Cancer Centers of Nevada, Las Vegas, NV USA; 4 Princess Margaret Cancer Centre, Toronto, Ontario Canada; 5 Yale Cancer Center, Yale University, New Haven, CT USA; 6 Memorial Sloan Kettering Cancer Center, New York, NY USA; 7 Department of Medicine Weill Cornell Medical College, New York, NY USA; 8 The Angeles Clinic and Research Institute, a Cedars-Sinai affiliate, Los Angeles, CA USA; 9 HonorHealth Research Institute, Scottsdale, AZ USA; 10 Karolinska University Hospital, Stockholm, Sweden; 11 Drug Research Unit Ghent, Ghent University Hospital, Ghent, Belgium; 12 University Hospital Southampton NHS Trust and University of Southampton, Southampton, UK; 13 Department of Medicine (Oncology), Stanford University, Stanford, CA USA; 14 University Medical Center Mainz, Mainz, Germany; 15 West German Cancer Center, Department of Medical Oncology, University Hospital Essen, University Duisburg-Essen, Essen, Germany; 16 Massachusetts General Hospital Cancer Center, Boston, MA USA; 17 Sarah Cannon Research Institute, Nashville, TN USA; 18 Vall d’Hebron Institute of Oncology (VHIO), Barcelona, Spain; 19 Columbia University Herbert Irving Comprehensive Cancer Center, New York, NY USA; 20 ZAS Ziekenhuizen, Oncology Center Antwerp (OCA) Campus Sint-Augustinus, Antwerp, Belgium; 21 National Center for Tumor Diseases, University Hospital of Heidelberg, Heidelberg, Germany; 22 Dana-Farber Cancer Institute and Harvard Medical School, Boston, MA USA; 23 University of Washington School of Medicine, Seattle, WA USA; 24 CHU Liege and Liege University, Liege, Belgium; 25 Department of Oncology, Uppsala University Hospital, Uppsala, Sweden; 26 UCSF Helen Diller Family Comprehensive Cancer Center, San Francisco, CA USA; 27 Netherlands Cancer Institute, Amsterdam, The Netherlands; 28 Earle A. Chiles Research Institute, Providence Cancer Institute, Portland, OR USA; 29 Lungenfachklinik Immenhausen, Immenhausen, Germany; 30 The Ottawa Hospital Cancer Centre, Ottawa, Ontario Canada; 31 University Medical Center Utrecht, Utrecht, Netherlands; 32 University of Oklahoma Health Sciences Center, Oklahoma City, OK USA; 33 University of Navarra and Instituto de Investigacion Sanitaria de Navarra and CIBERONC, Pamplona, Spain; 34 Genentech, Inc., South San Francisco, CA USA; 35 Stork Therapeutics, San Francisco, CA USA; 36 Bluejay Therapeutics, San Mateo, CA USA; 37 TRON Translational Oncology at the University Medical Center of the Johannes Gutenberg University Mainz, Mainz, Germany; 38 BioNTech, Mainz, Germany; 39 Helmholtz Institute for Translational Oncology Mainz (HI-TRON Mainz) by DKFZ, Mainz, Germany; 40 Department of Medicine-Medical Oncology, University of Colorado Cancer Center, Denver, CO USA; 41 Present Address: Seagen, Inc., Bothell, WA USA; 42 Present Address: Gilead Sciences, Foster City, CA USA; 43 Present Address: Artera, Inc., Los Altos, CA USA; 44 Present Address: AbbVie, Inc., Santa Clara, CA USA

**Keywords:** Cancer immunotherapy, Drug development

## Abstract

Effective targeting of somatic cancer mutations to enhance the efficacy of cancer immunotherapy requires an individualized approach. Autogene cevumeran is a uridine messenger RNA lipoplex-based individualized neoantigen-specific immunotherapy designed from tumor-specific somatic mutation data obtained from tumor tissue of each individual patient to stimulate T cell responses against up to 20 neoantigens. This ongoing phase 1 study evaluated autogene cevumeran as monotherapy (*n* = 30) and in combination with atezolizumab (*n* = 183) in pretreated patients with advanced solid tumors. The primary objective was safety and tolerability; exploratory objectives included evaluation of pharmacokinetics, pharmacodynamics, preliminary antitumor activity and immunogenicity. Non-prespecified interim analysis showed that autogene cevumeran was well tolerated and elicited poly-epitopic neoantigen-specific responses, encompassing CD4^+^ and/or CD8^+^ T cells, in 71% of patients, most of them undetectable at baseline. Responses were detectable up to 23 months after treatment initiation. CD8^+^ T cells specific for several neoantigens constituted a median of 7.3% of circulating CD8^+^ T cells, reaching up to 23% in some patients. Autogene cevumeran-induced T cells were found within tumor lesions constituting up to 7.2% of tumor-infiltrating T cells. Clinical activity was observed, including one objective response in monotherapy dose escalation and in two patients with disease characteristics unfavorable for response to immunotherapy treated in combination with atezolizumab. These findings support the continued development of autogene cevumeran in earlier treatment lines. ClinicalTrials.gov registration: NCT03289962.

## Abstract (web summary)

In this phase 1 trial, patients with locally advanced or metastatic solid tumors were treated with the individualized mRNA neoantigen-specific immunotherapy (iNeST) autogene cevumeran alone or in combination with the anti-PD-L1 agent atezolizumab, showing long-lasting neoantigen-specific immune responses and preliminary clinical activity, supporting further development of this therapeutic approach.

## Main

Cancer immunotherapy with checkpoint inhibitors (CPIs) has advanced the practice of oncology and become a standard of care across many cancer types. Despite these advancements, many patients do not have durable response. Additional approaches to promote CPI-mediated anticancer immunity could meet this therapeutic need^1^. The mode of action of CPIs is based on reinvigorating preexistent tumor-recognizing T cells^2^. The clinical efficacy of CPIs has been associated with promoting T cells directed against somatic cancer mutations^3–6^. Cancer mutations may give rise to neoantigens of exquisite cancer cell specificity that are not expressed in healthy tissue and therefore not subject to central tolerance.

Only a small fraction of potentially immunogenic cancer mutations give rise to spontaneously occurring neoantigen-specific T cells on which CPIs can act^7,8^. Vaccination with somatic cancer mutations is an attractive approach to prime neoantigen-specific T cells de novo or amplify preexisting ones and may contribute to improved efficacy of CPIs^9,10^.

Given that antigen-presenting human leukocyte antigen (HLA) molecules are highly polymorphic and that the vast majority of tumor neoantigens arise from patient-specific mutations rather than alterations shared across patients, effective targeting of somatic cancer mutations through vaccination requires an individualized approach^11,12^. Early clinical trials in small cohorts of patients with cancer showed the feasibility, safety, tolerability and induction of neoantigen-specific T cell responses with different vaccine platforms including antigen-encoding mRNA^13–17^.

We have developed autogene cevumeran, an individualized neoantigen-specific immunotherapy (iNeST), composed of up to two 5′-capped single-stranded uridine-based mRNA molecules (together encoding up to 20 neoantigens per patient) linked by short glycine- and serine-rich linkers and encapsulated in a lipoplex formulation (RNA–LPX). The neoantigen candidates are identified by a computational pipeline that uses next-generation sequencing data derived from blood and tumor biopsy for mutation calling and for prediction of each patient’s unique composition of potentially T cell immunity-inducing neoantigens^17^. The RNA backbone is designed for optimized translational performance of the coding sequence in human dendritic cells (DCs)^18–20^ and for augmented antigen presentation on HLA class I and II molecules^21^. The RNA–LPX is intravenously administered to target DCs residing in lymphoid compartments, including the spleen^22^. The RNA–LPX technology couples antigen delivery with co-stimulation through Toll-like receptor (TLR)-mediated, type 1 interferon-driven antiviral immune mechanisms and results in profound expansion of antigen-specific T cells as shown in a phase 1/2 trial in patients with advanced melanoma and nonmutated, tumor-associated self-antigens^22–24^.

We recently reported the results of an investigator-initiated, phase 1 trial of post-surgery adjuvant treatment of patients with resected pancreatic ductal adenocarcinoma (PDAC) with autogene cevumeran and the anti-PD-L1 CPI atezolizumab combined with standard-of-care adjuvant chemotherapy, showing substantial induction of high-magnitude, polyfunctional neoantigen-specific CD8^+^ effector T cells with longevity markers in a fraction of patients with cancer, which correlated with delayed PDAC recurrence^25,26^. The design of the study^25^ and other ongoing trials with autogene cevumeran were informed by the first-in-human phase 1 trial (NCT03289962), from which we report a non-prespecified interim analysis here. The ongoing study evaluated the feasibility, safety, tolerability, immunogenicity and preliminary antitumor activity of autogene cevumeran as monotherapy and in combination with atezolizumab. The primary objective was safety and tolerability of autogene cevumeran as monotherapy and in combination with atezolizumab. Exploratory objectives included the characterization of the pharmacokinetic and pharmacodynamic profile of autogene cevumeran as well as preliminary antitumor activity and immunogenicity.

## Results

### Patients, treatment and feasibility

This ongoing trial enrolled patients with cancer and locally advanced, metastatic or recurrent incurable malignancies between 21 December 2017 and 12 January 2022.

**Fig. 1:**
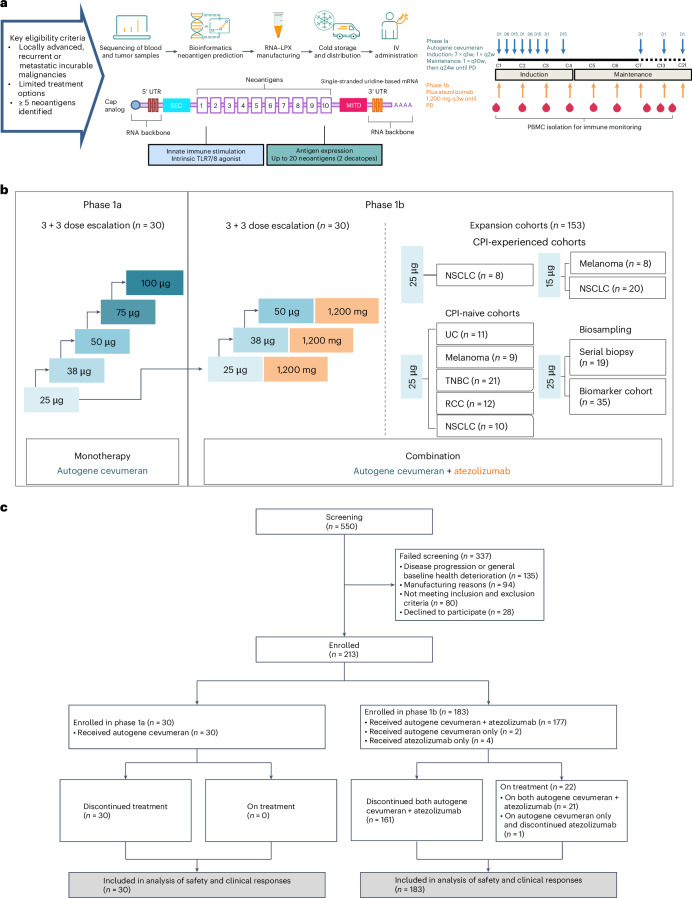
Phase 1 study design and patient disposition. **a**, Patients with locally advanced, recurrent or metastatic incurable tumors with at least five neoantigens and with limited treatment options were eligible. Autogene cevumeran: up to 20 tumor neoantigens identified by sequencing blood and tumor tissue were cloned into two RNA molecules (up to 10 neoantigens each) with backbones comprising a 5′-cap analog, 5′- and 3′-untranslated region (UTR) and poly(A) tail optimized for stability and translational efficiency. Neoantigens are flanked by an N-terminal SEC and an HLA class I trafficking domain (MITD, transmembrane and cytoplasmic domain of HLA class I) to ensure optimal antigen presentation and immunogenicity. For phase 1a, patients were treated with eight doses of autogene cevumeran administered intravenously over the first 9 weeks (induction), followed by periodic booster doses (maintenance) until disease progression. In phase 1b, autogene cevumeran was combined with 1,200 mg atezolizumab administered intravenously on day 1 of the combination regimen and given every 3 weeks until disease progression. **b**, Patients in phase 1a received autogene cevumeran in dose escalation cohorts ranging from 25 μg to 100 μg. In phase 1b, autogene cevumeran (25 μg to 50 μg as indicated) was combined with 1,200 mg atezolizumab in a dose escalation part, indication-specific expansion cohorts including a serial biopsy and a biomarker cohort. In the biomarker cohort, 16 of 35 patients received autogene cevumeran as monotherapy for the induction course; atezolizumab was added for the maintenance course. Patients in both cohorts underwent biopsy, leukapheresis and blood draws before and during treatment for immune monitoring. **c**, Participant flow diagram. The number of participants screened, enrolled, treated, discontinued and analyzed is shown for phase 1a and 1b. C1, C7, C13 and C21, cycle 1, 7, 13 and 21; D1, D8 and D15, day 1, 8 and 15; q1w, q2w, q3w, q10w and q24w, once every 1, 2, 3, 10 and 24 week(s).

Patients in phase 1a received autogene cevumeran as monotherapy in dose escalation cohorts ranging from 25 µg to 100 µg (Fig. 1a,b) per the schedule described in Methods. The dose range was informed by previous phase 1/2 clinical experience with another cancer vaccine derived from the same uridine-based RNA–LPX technology platform that encoded nonmutated shared tumor-associated antigens^24^. Patients in phase 1b received autogene cevumeran in combination with atezolizumab in dose escalation cohorts evaluating 25 µg, 38 µg and 50 µg of autogene cevumeran, indication-specific dose expansion cohorts, a serial biopsy expansion cohort or a biomarker substudy (Fig. 1b).

A total of 550 patients were screened for participation in the cohorts reported in this paper. We evaluated blood and tissue from 548 patients submitted for manufacturing autogene cevumeran. Overall, 512 patients submitted tissue specimens with sufficient tumor content, 483 patients (94.3%) had sufficient DNA and RNA isolated from specimens, and successful sequencing reactions were completed on blood and tumor tissue specimens from 473 patients. Of the 455 patients who remained in screening up to this point, the computational pipeline identified 5 or more neoantigens (eligibility criterion) from 448 patients (98.5%). Of these, 367 patients (81.9%) had at least 20 neoantigens identified.

Patients were excluded during screening at various time points for the following reasons: disease progression or general baseline deterioration (*n* = 135), reasons related to manufacturing (*n* = 94), not meeting inclusion and exclusion criteria (*n* = 80), or declining to participate (*n* = 28), in parallel to designing and producing autogene cevumeran. Manufacturing feasibility was shown with a best-case turnaround time (TAT) from reception and approval of a complete sample set to the end of manufacturing of 28 days. Overall, 213 patients were enrolled across 33 sites in North America and Europe between 21 December 2017 and the clinical data cutoff of 14 January 2022. Thirty patients were enrolled in phase 1a and 183 in phase 1b (Fig. 1b,c). The majority of enrolled patients had unfavorable prognostic baseline characteristics and had received multiple lines of previous systemic cancer therapy (including CPI), had multiple sites of metastatic disease, had elevated C-reactive protein and had tumors with negative or low PD-L1 scores (Extended Data Tables 1 and 2).

### Primary outcome: safety and tolerability

Two hundred and thirteen patients who received at least one dose of autogene cevumeran or atezolizumab were included in the safety analysis to assess the primary outcome. Nine (30.0%) of the 30 patients treated with autogene cevumeran monotherapy and 47 (25.7%) of the 183 patients treated in combination with atezolizumab discontinued study treatment owing to disease progression at or before the first tumor assessment at week 7 and before completing the induction course of autogene cevumeran. Thirteen (43.3%) patients in the monotherapy dose escalation crossed over to combination treatment (Supplementary Table 1).

**Fig. 2:**
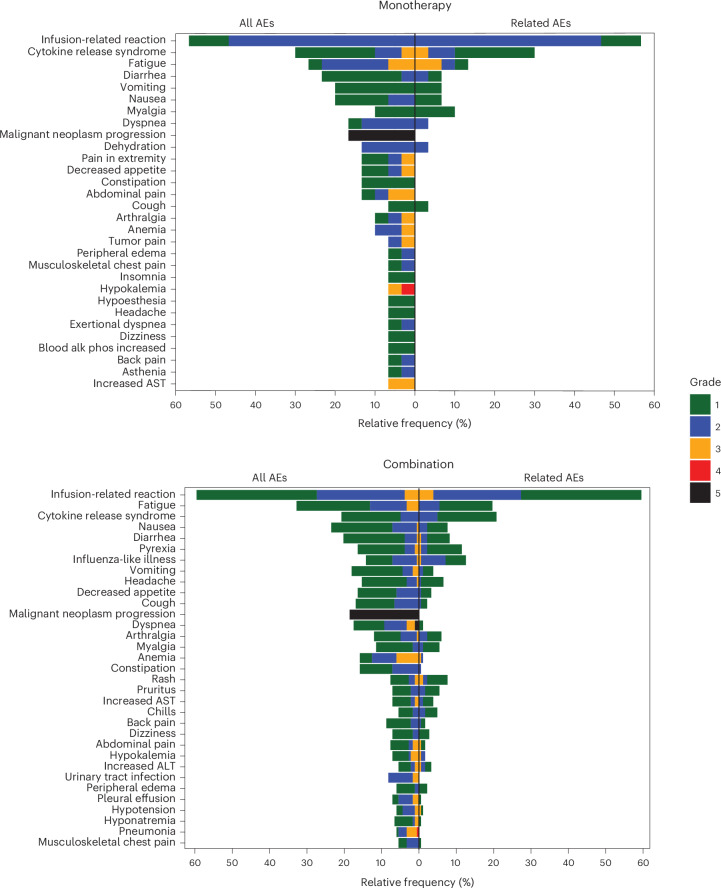
Safety and tolerability data of patients with advanced disease who received autogene cevumeran with or without atezolizumab. AEs occurring in ≥5% of patients are presented as all AEs and AEs related to treatment by grade. AEs related to treatment AEs were graded in line with the NCI CTCAE, v.5.0. Alk-phos, alkaline phosphatase; AST, aspartate aminotransferase; ALT, alanine aminotransferase.

Twenty-seven (90%) patients treated with autogene cevumeran monotherapy had treatment-related adverse events (TRAEs), of which 3 patients experienced a grade 3 adverse event (AE; 1 event of grade 3 cytokine release syndrome (CRS) and 2 events of grade 3 fatigue) with no grade 4 and 5 TRAEs observed (Fig. 2 and Table 1). No AEs or TRAEs led to treatment withdrawal. One dose-limiting toxicity (DLT) of grade 3 CRS was observed with 100 µg autogene cevumeran monotherapy. The event was resolved with supportive care, and the patient recovered and continued study treatment at a reduced dose until disease progression at day 82 (Table 1 and Supplementary Table 2).

**Table 1:** Safety overview for patients treated with autogene cevumeran as monotherapy and in combination with atezolizumab

Phase 1a safety overview						
AE, *n* (%)	Autogene cevumeran monotherapy dose					
	25 µg (*n* = 12)	38 µg (*n* = 5)	50 µg (*n* = 4)	75 µg (*n* = 8)	100 µg (*n* = 1)	All patients (*n* = 30)
All-grade AEs, any cause	12 (100)	5 (100)	4 (100)	8 (100)	1 (100)	30 (100)
Grade 3 AEs	4 (33.3)	0	2 (50)	4 (50)	1 (100)	11 (36.7)
Grade 4 or 5 AEs	2 (16.7)	0	2 (50)	3 (37.5)	0	7 (23.3)
Treatment-related AEs	10 (83.3)	4 (80.0)	4 (100)	8 (100)	1 (100)	27 (90.0)
Grade 3 TRAEs	0	0	1 (25.0)	1 (12.5)	1 (100)	3 (10.0)
Grade 4 or 5 TRAEs	0	0	0	0	0	0
DLT	0	0	0	0	1 (100)	1 (3.3)
Serious AEs	5 (41.7)	0	2 (50.0)	4 (50.0)	1 (100)	12 (40.0)
AEs leading to any treatment withdrawal	0	0	0	0	0	0
TRAEs leading to any treatment withdrawal	0	0	0	0	0	0

Phase 1b safety overview					
AE, *n* (%)	Autogene cevumeran dose + atezolizumab 1,200 mg				
	15 µg (*n* = 28)	25 µg (*n* = 135)	38 µg (*n* = 11)	50 µg (*n* = 9)	All patients (*n* = 183)
All-grade AEs, any cause	28 (100)	135 (100)	11 (100)	9 (100)	183 (100)
Grade 3 AEs	12 (42.9)	63 (46.7)	5 (45.5)	5 (55.6)	85 (46.4)
Grade 4 or 5 AEs	7 (25)	33 (24.4)	1 (9.1)	3 (33.3)	44 (24)
Treatment-related AEs	21 (75.0)	125 (92.6)	11 (100)	8 (88.9)	165 (90.2)
Grade 3 TRAEs	2 (7.1)	24 (17.8)	3 (27.3)	3 (33.3)	32 (17.5)
Grade 4 or 5 TRAEs	0	3 (2.2)	0	0	3 (1.6)^a^
DLT	0	0	0	0	0
Serious AEs	11 (39.3)	76 (56.3)	3 (27.3)	6 (66.7)	96 (52.5)
AEs leading to any treatment withdrawal	1 (3.6)	22 (16.3)	1 (9.1)	2 (22.2)	26 (14.2)
TRAEs leading to any treatment withdrawal	0	10 (7.4)	0	1 (11.1)	11 (6.0)

Data are reported as *n* (%) of patients who received at least one dose of autogene cevumeran or atezolizumab. Events were classified according to the CTCAE, v.5.0.

^a^Two events of grade 4 pneumonitis and one event of grade 4 systemic immune activation.

No new signals and no DLTs were observed when autogene cevumeran was combined with atezolizumab (Fig. 2). Three grade 4 or 5 TRAEs (1 event each of grade 4 pancreatitis, grade 4 systemic immune activation and grade 5 pneumonitis) were observed, and 11 patients (6%) in phase 1b discontinued study treatment owing to TRAEs (Table 1). The severity, frequency and clinical nature of immune-mediated AEs were generally consistent with atezolizumab monotherapy experience (Supplementary Table 3)^27^.

**Extended Data Fig. 1:**
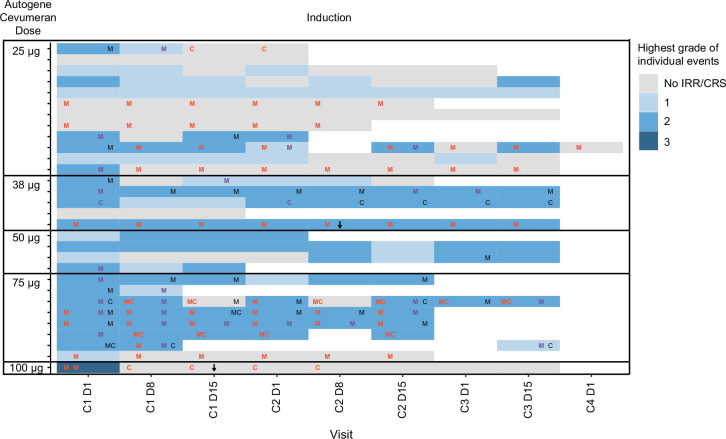
Systemic reactions during the induction course of vaccination in phase 1a (autogene cevumeran monotherapy) by dose. Red text represents concomitant medications given before or after autogene cevumeran administration. Purple represents concomitant medications given for the treatment of adverse events. Black text represents concomitant medications given on the same day as autogene cevumeran administration but not indicated as pre or post dose administration or for treatment of adverse events. IRR and CRS events were classed according to the Common Terminology Criteria for Adverse Events, version 5.0. C, corticosteroids; CRS, cytokine release syndrome; IRR, infusion-related reaction; M, meperidine; down arrow, dose reduction.

The most common TRAEs were infusion-related reaction (56.7% and 59.6% for monotherapy and combination therapy, respectively), CRS (30% and 20.8%) and influenza-like illness (3.3% and 12.6%) (Fig. 2 and Supplementary Table 2). These TRAEs were predominantly grade 1 or 2 and were consistent with systemic reactions after infusion, typically presenting as fever, chills and rigor, and nausea (Supplementary Table 2), which were transient and readily manageable. Antipyretics, meperidine and, in some cases, corticosteroids as well as dose reductions of autogene cevumeran were used for management or prophylaxis of grade 2 or 3 systemic reactions (Extended Data Fig. 1). A trend toward increased use of concomitant medications to manage systemic reactions at higher doses of autogene cevumeran monotherapy was observed, suggesting possible dose-dependency of systemic reactions.

**Extended Data Fig. 2:**
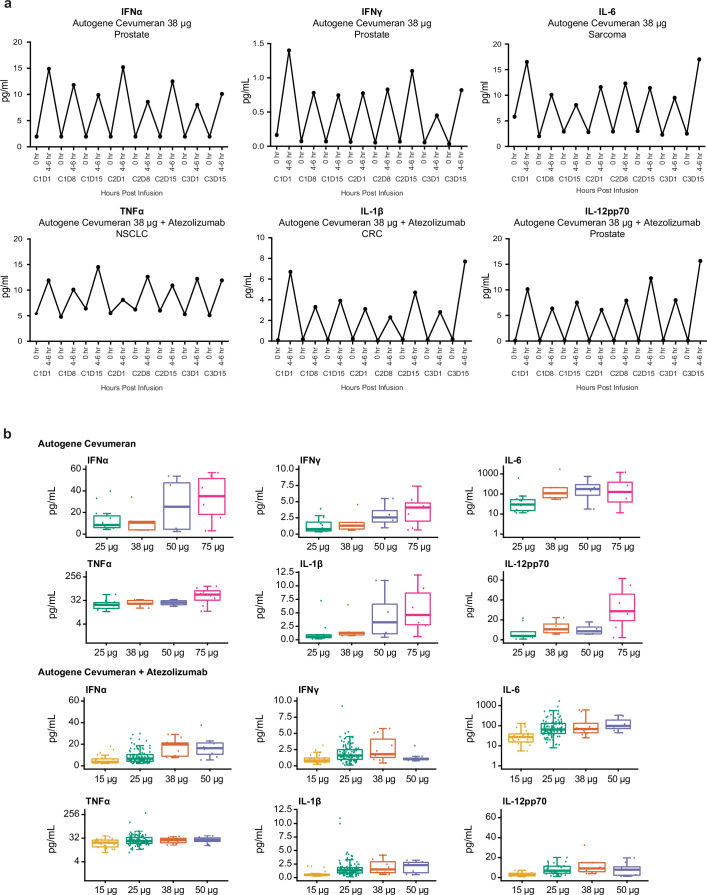
Changes in immune-related cytokine levels prior to and during treatment. **a**, Cytokine profiles of individual patients during the induction phase. **b**, Peak plasma cytokine levels (4–6 h after autogene cevumeran injection) for patients treated with autogene cevumeran monotherapy in phase 1a or in combination with atezolizumab in phase 1b. Boxes show the 25th to 75th quantiles with lines representing medians from timepoints 4–6 h after infusion from multiple vaccinations; whiskers show minimum to maximum values; each dot represents one patient value per dose level. Sample sizes for each cytokine analysis were: IFNα *n* = 28 (Ph1a), *n* = 137 (Ph1b); IFNγ *n* = 28 (Ph1a), *n* = 147 (Ph1b); IL-6 *n* = 28 (Ph1a), *n* = 144 (Ph1b); TNFα *n* = 29 (Ph1a), *n* = 144 (Ph1b); IL-1β *n* = 28 (Ph1a), *n* = 141 (Ph1b); IL-12p70 *n* = 23 (Ph1a), *n* = 63 (Ph1b).

Transient, dose-level-dependent increases in plasma interferon alpha (IFNα), interferon gamma (IFNγ), interleukin 6 (IL-6), interleukin 12 (IL-12p70), interleukin-1 beta (IL-1β) and tumor necrosis factor (TNF) levels were observed 4–6 h after infusion of autogene cevumeran with return to baseline levels both as monotherapy and in combination with atezolizumab (Extended Data Fig. 2).

Dose-level decisions were made based on aggregate data guided by safety and tolerability considerations. The dose of autogene cevumeran in combination with atezolizumab was not increased beyond 50 μg based on observed trends for dose-dependent increases in systemic reactions in association with increased levels of inflammatory cytokines (Extended Data Figs. 1 and 2). On the basis of these considerations, a 25 μg dose was selected for the dedicated CPI-naive dose expansion cohorts. In parallel, expansion cohorts enrolling CPI-experienced patients were opened at 25 μg and 15 μg.

### Characterization of T cell responses

**Fig. 3:**
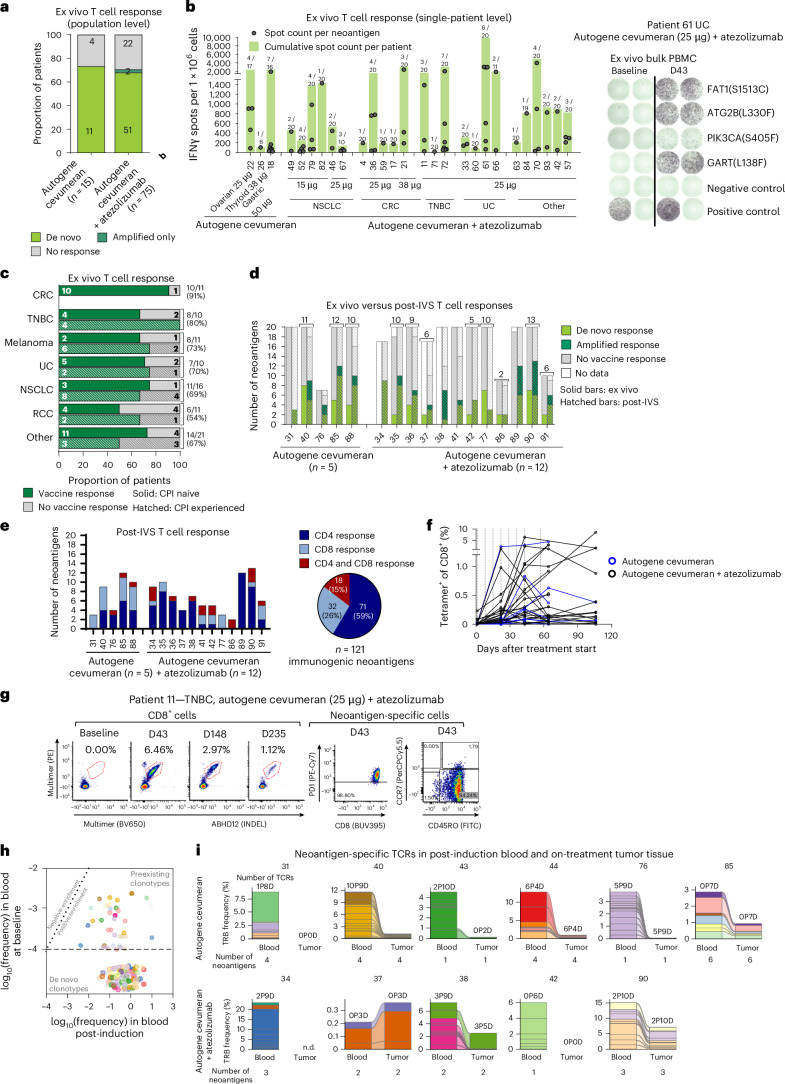
Autogene cevumeran induced de novo CD4^+^ and CD8^+^ T cell responses against multiple neoantigens in the blood and tumor of most patients. **a**,**b**, Ex vivo IFNγ ELISpot data of 90 patients and 1,404 neoantigens. **a**, Proportion of patients with autogene cevumeran-induced T cell responses. **b**, Magnitude of autogene cevumeran-induced neoantigen-specific T cells in patients with available bulk PBMC ELISpot data on single neoantigens (left) and example patient (right). Numbers above the bars: numbers of neoantigens inducing a T cell response among all encoded neoantigens. Negative control: PBMCs with medium; positive control: anti-CD3 antibody**. c**, Autogene cevumeran-induced T cell responses per indication and CPI status. ‘Other’ includes indications with at most five patients. The numbers on the right show the immune response rate per indication. **d**,**e**, IFNγ ELISpot analysis after IVS of enriched CD4^+^ and CD8^+^ T cells with autogene cevumeran neoantigens in 17 patients and 234 evaluable neoantigens. **d**, Autogene cevumeran-induced neoantigen-specific T cell responses detected after IVS and ex vivo IFNγ ELISpot (*n* = 15). The numbers above the bars are the total numbers of immunogenic neoantigens detected by either assay. **e**, Phenotype of T cell responses at the patient level (bar chart) and the population level (pie chart). **f**, Kinetics of neoantigen-specific CD8^+^ T cells shown using pHLA multimer staining of PBMCs from 22 patients. The dotted lines indicate autogene cevumeran administration. **g**, Left, de novo induced CD8^+^ T cell response of patient 11 against an autogene cevumeran-encoded INDEL, followed for 8 months and, right, memory phenotyping with PD-1 expression of the pHLA multimer-stained T cells. **h**, Frequency of neoantigen-specific TCRs in circulation. **i**, Cumulative frequency of neoantigen-specific TCRs in CD8^+^ cells in post-induction blood and on-treatment tumor tissues collected between 35 and 56 days after treatment start (147 days for patient 38; *n* = 10 biomarker cohort patients). The CDR3 beta sequences were used for tracking in bulk profiling data. The colors represent distinct neoantigens. Numbers above the bars: total numbers of neoantigen-specific TCRs; numbers below the bars: numbers of corresponding neoantigens. D, de novo; n.d., not done; P, preexisting; TRB, T cell receptor β; FITC, fluorescein isothiocyanate.

Neoantigen-specific T cells were analyzed at baseline and after the induction phase by two IFNγ ELISpot assay approaches (Supplementary Table 4). An ex vivo ELISpot assay without previous in vitro expansion, capturing high-magnitude, late-differentiated T cell responses, was performed for 90 patients, comprehensively testing all neoantigens encoded by autogene cevumeran across these patients. De novo induction or amplification of T cell responses against at least one of their autogene cevumeran-encoded neoantigens was detected in 64 of 90 patients (71%)—at similar frequencies in the monotherapy (11 of 15 patients, 73%) and combination cohorts (53 of 75 patients, 71%; Fig. 3a). Almost all patients with T cells recognizing autogene cevumeran neoantigens had de novo induced responses (62 of 64 patients, 97%); 8 of these patients had additional preexisting T cell responses amplified by autogene cevumeran, indicating that the vast majority of predicted strong neoantigens do not spontaneously activate T cells.

Ex vivo-detectable immune responses were observed across all tested tumor types in both CPI-naive and CPI-experienced patients and were most frequent in microsatellite stable colorectal cancer (CRC; 10 of 11 patients, 91%), triple-negative breast cancer (TNBC; 8 of 10 patients, 80%) and melanoma (8 of 11 patients, 73%) (Fig. 3b,c).

The number of neoantigens per patient that induced an ex vivo-detectable immune response varied between individuals (median, 2; range, 1–8), as did the magnitude of the T cell response per single neoantigen (Fig. 3b). Neither appeared to be affected by the dose of autogene cevumeran or whether it was administered as monotherapy or in combination.

**Extended Data Fig. 3:**
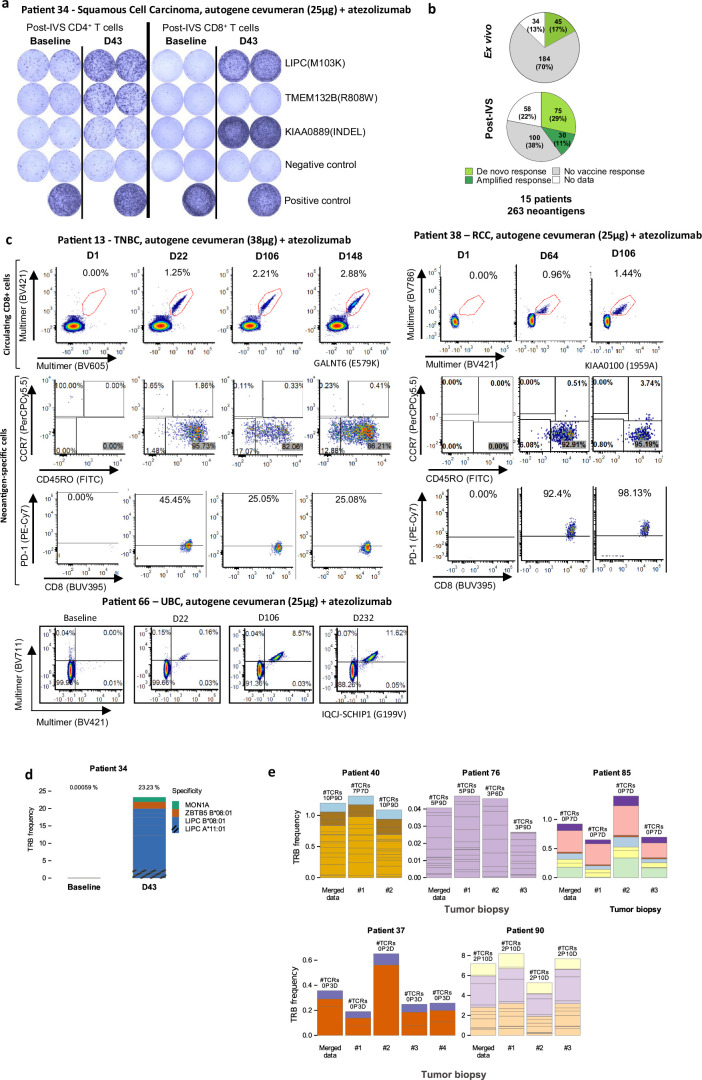
Autogene cevumeran-induced T-cell responses in individual patients. **a**, Concomitant autogene cevumeran-amplified CD4^+^ and *de novo* CD8^+^ T-cell response against LIPC (M103K) and autogene cevumeran-induced CD4^+^ or CD8^+^ responses against two other neoantigens determined by post-IVS IFNγ-ELISpot in a combination-treated patient with SCC. **b**, Comparison of IFNγ ELISpot detected T-cell responses against 263 autogene cevumeran-encoded neoantigens administered across 15 patients for whom both *ex vivo* and post-IVS ELISpot was performed. 205 neoantigens were evaluable for post-IVS and 229 for ex vivo ELISpot **c**, Kinetics, memory phenotype and PD-1 expression of selected autogene cevumeran-induced neoantigen-specific CD8^+^ T-cell responses from three patients. PBMCs stained with neoantigen-specific pHLA multimers (first and fourth rows). pHLA multimer+ cells stained for CD45RO and CCR7 (second row) or CD279 (PD-1, third row). **d**, Neoantigen-specific CD8^+^ TCRs identified from blood post-induction for patient 34. Specificity of selected TCRs was tested *in vitro* against patient-specific neoantigen/HLA combinations. Freq **e**, Neoantigen-specific TCRs identified in multiple on-treatment tumor biopsies. CDR3 beta sequences were used as molecular identifiers to track the neoantigen-specific TCRs in bulk TCRβ (TRB) profiling data from multiple on-treatment tumor biopsies of five patients from the biomarker cohort. The cumulative frequency of neoantigen-specific TCRs in all biopsies (‘merged data’) and for each biopsy is shown for each patient. Each color represents a different neoantigen target. Numbers above the bar charts show the number of neoantigen-specific TCRs tracked in each sample for each patient. D, *de novo*; IVS, *in vitro* stimulation; P, pre-existing; PBMC, peripheral blood mononuclear cells; TCR, T-cell receptor.

Blood samples from 17 patients from the biomarker substudy were analyzed with an ELISpot approach that tests CD4^+^ and CD8^+^ T cells separately after 2 weeks of in vitro stimulation (IVS) with the respective patients’ autogene cevumeran neoantigens. This analysis captures low-frequency, early-differentiated, highly proliferative T cells that may not be detectable ex vivo. All 17 patients showed autogene cevumeran-induced CD4^+^ and/or CD8^+^ T cell responses against at least two encoded neoantigens (median, 7; range, 2–13) that were mostly induced de novo (Fig. 3d; representative patient shown in Extended Data Fig. 3a). In total, post-IVS ELISpot identified T cell responses against 52% (121 of a total of 234) of autogene cevumeran neoantigens. The majority of these neoantigens were targeted by CD4^+^ T cells only (59%), 26% were recognized by CD8^+^ only (26%) and a smaller fraction was recognized concomitantly by CD4^+^ and CD8^+^ T cells (15%, Fig. 3e).

Fifteen patients were tested with both assays. In these patients, the post-IVS ELISpot detected more than twice as many autogene cevumeran-induced T cell responses than the ex vivo ELISpot (105 versus 45 neoantigens, respectively), representing 51% of the 205 evaluable neoantigens, including multiple responses in 4 patients (patients 31, 41, 76 and 89) without ex vivo-detectable T cells (Fig. 3d and Extended Data Fig. 3b). Patients in this subset had autogene cevumeran-induced and amplified T cells against a median of 6 neoantigens (range, 3–12), revealing that autogene cevumeran has a broader immunogenicity than that measured by ex vivo ELISpot (median, 2, and range, 2–8, in this subset).

Peptide–MHC class I (pMHC) multimer analysis of selected patients (*n* = 22) showed that neoantigen-specific CD8^+^ T cells typically became detectable around 3 weeks after starting vaccination, continued to expand in the first couple of months and, in a fraction of patients, reached up to single-digit percentages of circulating T cells (Fig. 3f). Autogene cevumeran-induced T cells directed against one of the neoantigens persisted for several months in a patient with bladder cancer (patient 66), which constituted >11% of circulating CD8 T cells at day 232 (Extended Data Fig. 3c). Neoantigen-specific CD8^+^ T cell responses were mainly of an effector memory phenotype (CD45RO^+^CCR7^−^), with high expression of PD-1 (Fig. 3g and Extended Data Fig. 3c).

**Extended Data Fig. 4:**
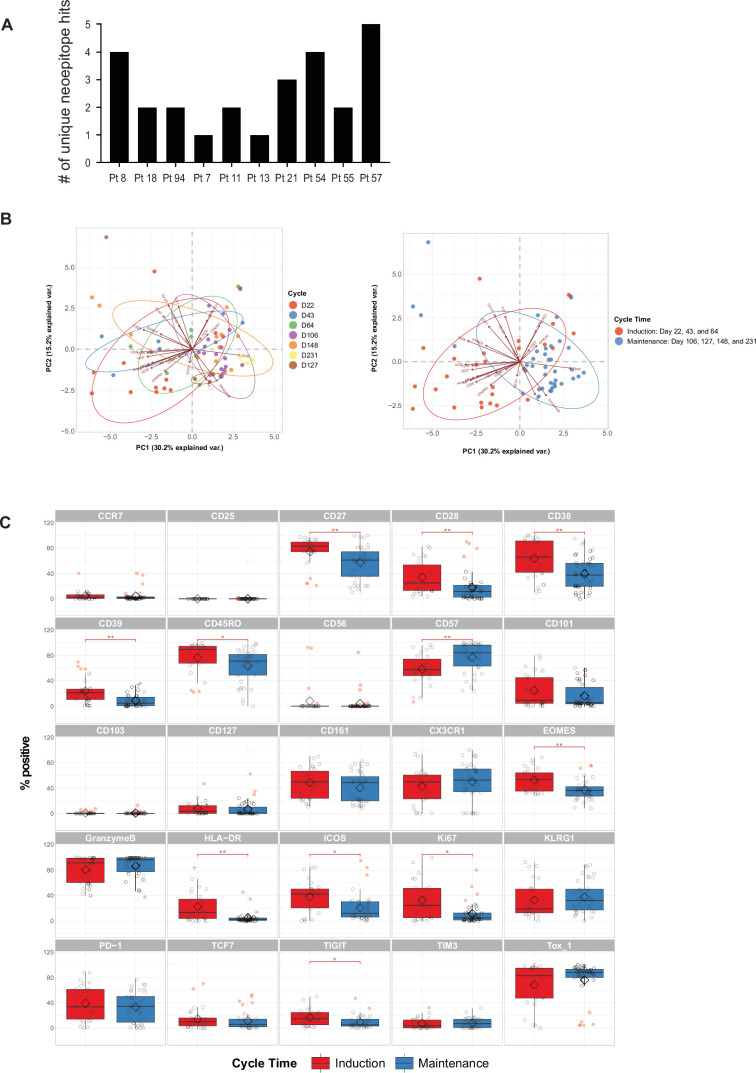
Deep profiling of neoantigen-specific CD8 T cells from longitudinal time points using mass cytometry (CyTOF). **a**, Number of unique neoepitope specificities detected in peripheral blood of 10 patients tested in mass cytometry analysis. 3 patients were from Ph1A (autogene cevumeran only) and 7 patients were from Ph1B (autogene cevumeran and atezo combination). **b**, Principal Component Analysis (PCA) of neoantigen-specific CD8 T cell populations identified across all 10 patients. PCA is based on phenotypic profiling (percent of neoantigen-specific CD8 T cells positive for all phenotypic markers assessed). Data points are colored based on time of collection (left plot) or segregated into early (red) or late (blue) time points (right plot). **c**, Frequency of expression for each individual markers on the neoantigen specific CD8 T cells compared between all early (red) pooled or late (blue) pooled time points (as described in b) (n = 27 for induction and n = 38 for maintenance data points). Data are presented as box whisker plots depicting the median, 25% (Q1) and 75% (Q3) quantiles, with the mean added in diamond shape. Minimum and maximum points are defined as Q1-1.5*IQR and Q3 + 1.5*IQR, respectively, where IQR is the inter-quantile range between Q1 and Q3. Any data point falling above or below the maximum or minimum points are marked in red circles. P values were calculated using two-sided Wilcoxon signed rank-sum test and are marked as: * P < 0.05, **P < 0.01. (Individual p values are CD27: 0.009; CD28: 0.009; CD38:0.009; CD39:0.004; CD45RO: 0.04; CD57:0.009; EOMES:0.0007; HLA-DR:0.004; ICOS:0.016; Ki67:0.01; TIGIT:0.03). P values were adjusted for multiple testing using the Benjamini-Hochberg method to control the false discovery rate.

To further characterize the activation profile and functional status of neoantigen-specific CD8 T cells over time, we conducted a longitudinal analysis using pMHC multimers in high-dimensional mass cytometry (CyTOF) on peripheral blood samples from patients (monotherapy *n* = 3 and combination therapy *n* = 7) (Extended Data Fig. 4a). Among these patients, we identified T cells reactive against 26 unique neoepitopes (with 1 to 5 reactivities per patient). We analyzed the phenotypes of neoantigen-specific T cells based on their detection in induction or maintenance cycles. Notably, all neoantigen-specific T cells showed time-dependent clustering (Extended Data Fig. 4b). Specifically, neoantigen-specific CD8 T cells showed a proliferative and early activation signature marked by elevated levels of Ki-67, HLA-DR, CD38 and ICOS at 22, 43 and 64 days after treatment initiation with autogene cevumeran. Neoantigen-specific CD8 T cells after ≥100 days of treatment, in contrast, underwent a transition from early- to late-differentiated effector phenotype marked by a decrease in CD27/CD28 expression and an increase in CD57 levels (Extended Data Fig. 4c).

We aimed to further understand the T cell response by cloning the T cell receptors (TCRs) of the CD8^+^ T cells most enriched after eight vaccination cycles. We identified 140 neoantigen-specific TCRs across 13 patients. These TCRs showed specific recognition of antigen-presenting cells when loaded with specific neoantigens as peptides or RNA. Autogene cevumeran-specific TCRs constituted a large portion of the post-vaccination circulating CD8^+^ T cell repertoires that reached up to double-digit cumulative frequencies (median, 7.3%; range, 0.2–23.2%), indicating strong expansion of neoantigen-specific T cells during the induction phase (Fig. 3g,h and Extended Data Fig. 3d). Notably, the majority of these TCRs were undetectable at baseline (99 of 140), indicating the priming of de novo T cells, which is consistent with our findings by ELISpot and pMHC multimer analysis (Fig. 3h). Responses were largely polyclonal with a median of two specific TCRs per neoantigen (range, 1–13), some with more than one HLA restriction per neoantigen (Fig. 3h and Supplementary Table 5; representative patient shown in Extended Data Fig. 3d). To assess whether poly-epitopic autogene cevumeran-induced T cells identified in the periphery home into tumor lesions, we investigated on-treatment tumor samples from 10 patients in which TCR discovery was performed. Almost all neoantigen-specific TCRs identified in peripheral blood were tracked in the autologous on-treatment tumor biopsies of 8 of 10 evaluable patients (75 of 89 TCRs), indicating that autogene cevumeran-induced neoantigen-specific T cells are capable of homing into the tumor. On-treatment tumor biopsies typically contained polyclonal TCR clonotypes specific to up to six autogene cevumeran-induced neoantigens, reaching frequencies of up to 7.2% (median, 1%; range, 0.04–7.2%) of infiltrating CD8^+^ TCR clonotypes (Fig. 3i, patient 90). In five patients, multiple biopsies from the same lesion for each patient were analyzed, showing high overlap between the detected neoantigen-specific TCRs in different samples (Extended Data Fig. 3e and Supplementary Table 5).

### Preliminary antitumor activity

**Fig. 4:**
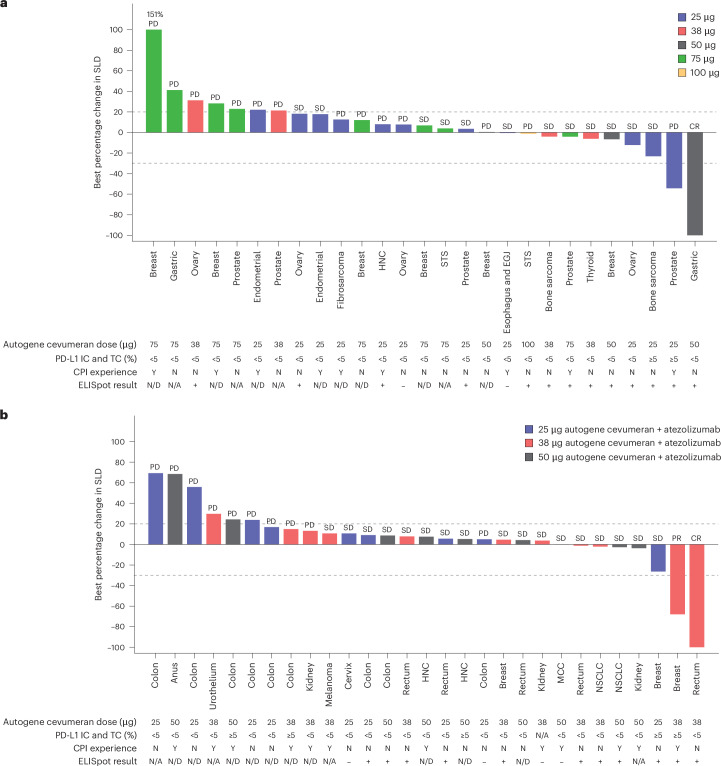
Antitumor activity of autogene cevumeran as monotherapy or in combination with atezolizumab in patients with advanced cancers in dose escalation cohorts. **a**,**b**, Clinical activity in patients in dose escalation cohorts with evaluable baseline and postbaseline measurements, assessed as the effect of autogene cevumeran as monotherapy (**a**) and in combination with atezolizumab (**b**) on target lesions per RECIST v.1.1 (*n* = 27 for phase 1a and *n* = 29 for phase 1b). ELISpot ‘+’ indicates that the patient had a neoantigen that indicated a positive response. HNC, head and neck cancer; EGJ, esophagogastric junction; MCC, Merkel cell carcinoma; N, no; N/A, not available; N/D, not done; SD, stable disease; STS, soft tissue sarcoma; Y, yes.

In the dose escalation cohorts, individual patients showed durable clinical benefit: a complete response (CR; gastric cancer) as best overall response (BOR) in the 50 µg autogene cevumeran monotherapy cohort and one partial response (PR) and a CR (breast and rectal cancer) in the 38 µg combination cohort (Fig. 4 and Supplementary Table 6).

**Extended Data Fig. 5:**
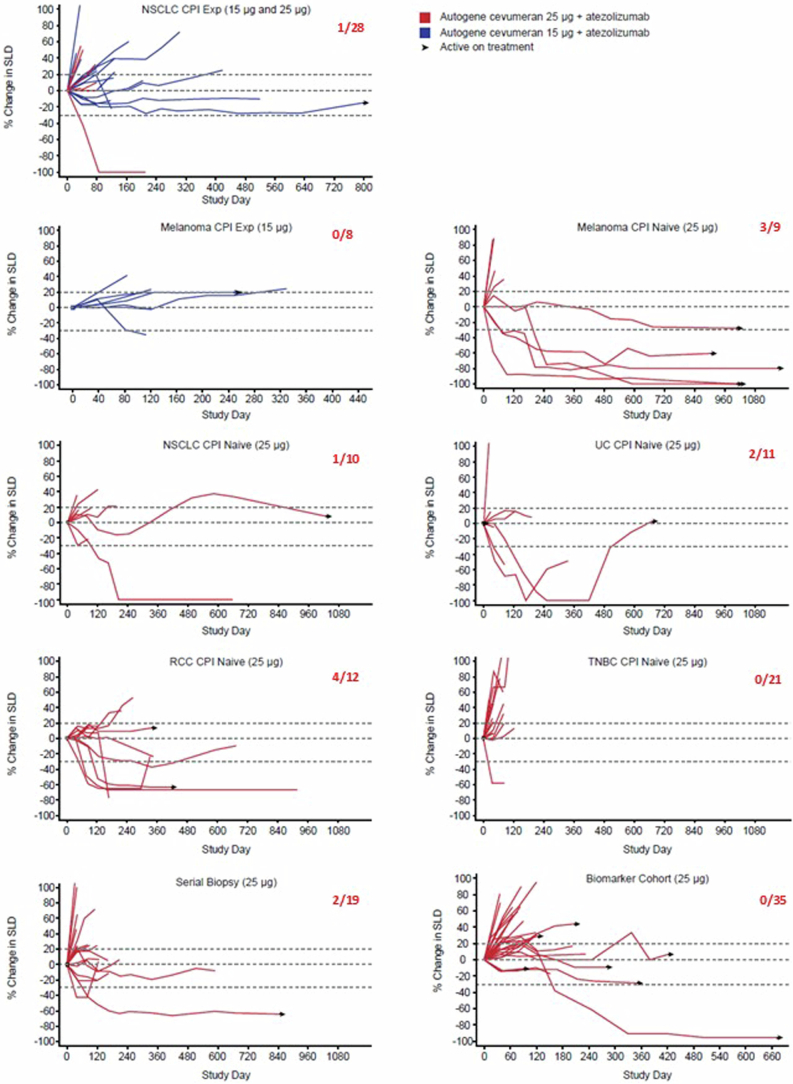
Anti-tumor activity of autogene cevumeran in combination with atezolizumab in patients with advanced cancers in expansion cohorts. Clinical activity observed in the expansion cohorts, assessed as the effect of autogene cevumeran and atezolizumab combination therapy on target lesions per RECIST 1.1. Numbers in red indicate the number of responses observed over the number of patients in each cohort. SLD, sum of longest diameters (of baseline tumors); CPI, checkpoint inhibitor. UC, Urothelial cancer; RCC, renal cell cancer; NSCLC, non–small cell lung cancer; RECIST v1.1, Response Evaluation Criteria in Solid Tumours version 1.1; TNBC, triple-negative breast cancer.

Further signal seeking for the autogene cevumeran–atezolizumab combination in CPI-experienced patients with non-small cell lung cancer (NSCLC, *n* = 28) or melanoma (*n* = 8) undertaken in phase 1b expansion cohorts (Extended Data Fig. 5) showed confirmed objective response in one patient with NSCLC (3.6%) (Supplementary Table 7), with 4 patients remaining on study treatment for >1 year.

CPI-naive melanoma (*n* = 9) and renal cell cancer (RCC; *n* = 12) expansion cohorts had an objective response rate (ORR) of 33.3%. The ORRs in the CPI-naive expansion cohorts in urothelial cancer (UC; *n* = 11), NSCLC (*n* = 10) and TNBC (*n* = 21) were 18.2%, 10.0% and 0%, respectively (Extended Data Fig. 5 and Supplementary Table 8). Seven patients (five with melanoma) continued to be followed for progression for 2 years, including three patients who continued treatment after initial progression of disease (Extended Data Fig. 5). However, these cohorts enrolled patients who had predominantly low or negative PD-L1 staining (<5% PD-L1 expression on tumor-infiltrating immune cells (IC) and tumor cells (TC) per SP142) (Table 1), thereby compromising direct comparison with historical efficacy benchmarks achieved with CPI monotherapy.

**Extended Data Fig. 6:**
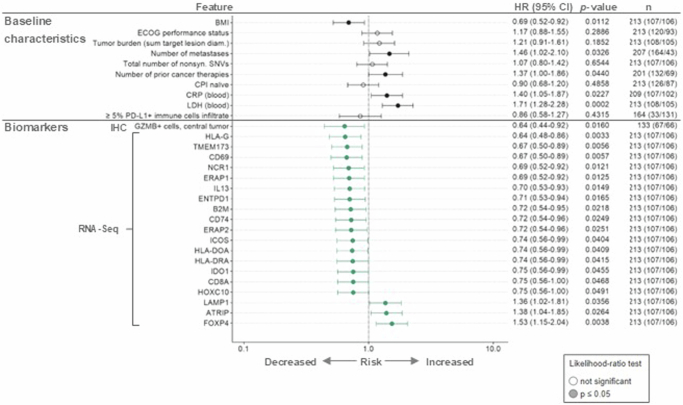
Impact of selected baseline factors on progression-free survival (PFS). Impact of selected baseline factors on PFS. Univariate Cox proportional hazard (CpxPH) analysis performed across patients with available PFS data. The forest plot shows the hazard ratios (HRs), that is, the exponentiated coefficients of the CoxPH model, with 95% confidence intervals as error bars, according to each single factor. Statistical significance was assessed by the two-sided likelihood-ratio test. P-values are based on the univariate likelihood-ratio test performed for a subset of selected baseline factors, thus p-values adjusted for multiple testing are not reported. BMI, body mass index; CPI, checkpoint inhibitor; CRP, C-reactive protein; ECOG, Eastern Cooperative Oncology Group; HR, hazard ratio; IHC, immunohistochemistry; LDH, lactate dehydrogenase; SNV, single-nucleotide variant.

Baseline characteristics and biomarkers of all patients with available progression-free survival (PFS) data (*n* = 213) were analyzed to identify factors associated with likelihood of disease progression (Extended Data Fig. 6). Patients with an increased number of metastases and previous cancer therapies and higher lactate dehydrogenase and C-reactive protein levels in blood at baseline were more likely to progress during the study. High tumor expression of markers for activated immune cell infiltrates (for example, *GZMB*, *CD69*, *NCR1* and *CD8A*), immune checkpoints (for example, *ICOS* and *IDO1*), antigen-presentation-related genes (for example, *HLA*, *B2M*, *ERAP1*, *ERAP2* and *CD74*) and other immune signaling genes were associated with a reduced risk of disease progression (Extended Data Fig. 6).

### Characterization of T cells in responding patients

**Extended Data Fig. 7:**
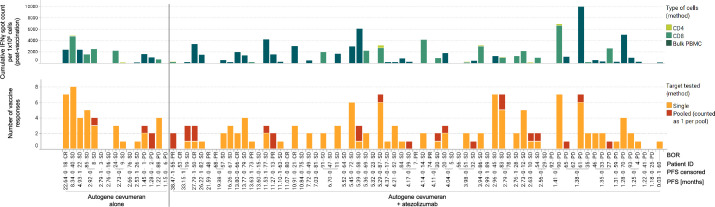
Correlation of clinical response and autogene cevumeran-induced T-cell response. Correlation of clinical best overall response (BOR) with de novo and amplified neoantigen-specific T-cell responses. Either the cumulative IFNγ spot count (fitness-normalized, per 1×106 cells) of all targets with autogene cevumeran response per type of cells assayed or the number of neoantigen-specific T-cell responses detected, both measured by ex vivo ELISpot assay post-vaccination, were correlated with clinical response. T-cell responses for monotherapy were measured after ≥7 vaccinations in patients treated with autogene cevumeran monotherapy in phase 1a or participants of the biomarker substudy not yet treated with atezolizumab. Participants treated with autogene cevumeran and atezolizumab are from phase 1b. Progression-free survival (PFS) censoring events were labelled with a 1.

Due to small sample sizes per tumor type, and the heterogeneity of the patient population and of the immunogenicity data, no conclusive analysis of correlation between clinical activity and autogene cevumeran-induced immune responses was feasible (Extended Data Fig. 7). We further characterized the immune responses and the disease course of one patient treated with monotherapy and three patients treated with the atezolizumab combination who had objective responses.

Patient 18, who had CPI-naive microsatellite instability-high, PD-L1-low, HER2+ gastroesophageal adenocarcinoma with a 1 cm target lesion in the liver and had been previously treated with three lines of systemic therapy, developed a CR after 8 doses of 50 µg autogene cevumeran monotherapy with a duration of response of 21 months. Patient 18 mounted strong poly-epitopic ex vivo-detectable de novo T cell responses to 7 of 16 neoantigens (Fig. 3b). For 5 of 7 neoantigens, we identified 12 CD8^+^ TCR clonotypes, which together constituted up to ~10% of the circulating CD8^+^ TCR repertoire and after 9 months were still at a frequency of over 7% (Fig. 4a, top). Against two neoantigens, CD4^+^ TCRs of lower frequency were detected (Fig. 4a, bottom). Five of the CD8^+^ TCR clonotypes and one of the CD4^+^ TCR clonotypes were specific for SVEP1 (R136C) that had shown the strongest response in ex vivo ELISpot.

**Fig. 5:**
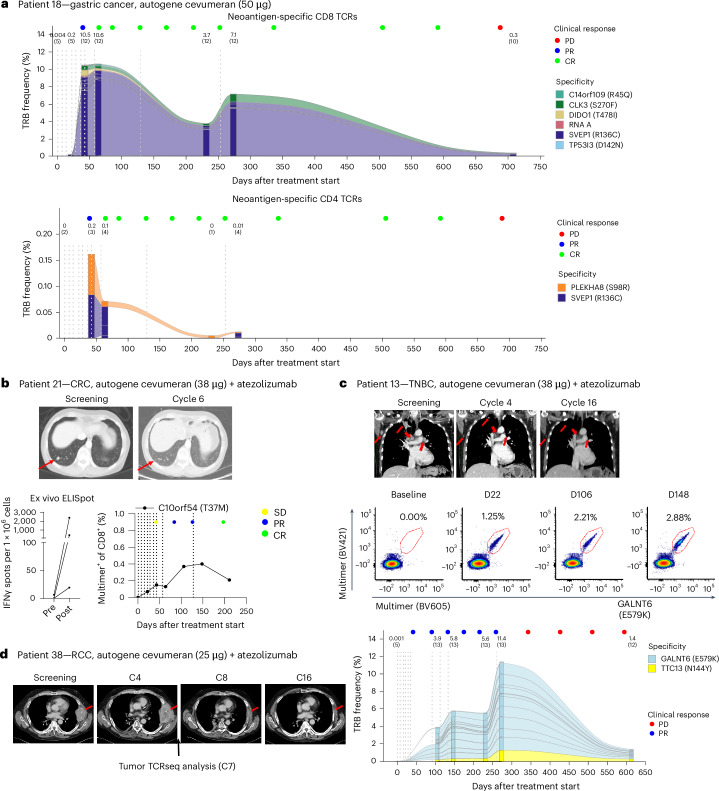
Patients with objective responses showed poly-epitopic autogene cevumeran-induced T cell responses. **a**, Neoantigen-specific TCRs isolated from longitudinal blood samples in patient 18. TRB CDR3 sequences of neoantigen-specific TCRs were tracked in bulk CD8^+^ (top) and CD4^+^ (bottom) TRB profiling data. The bar color represents different specificities. The vertical dashed lines represent vaccination time points. Cumulative frequency and number (in brackets) of neoantigen-specific TCRs are shown for each time point above the respective bar, above which colored dots represent evaluation of the clinical response at that time point. **b**–**d**, Lesions, indicated by red arrows, are shown before and after study treatment. Lung CT scans of patient 21 with CRC (top), with the magnitude of T cell responses to individual neoantigens detected by ex vivo IFNγ ELISpot (bottom left) and time-course analysis of a CD8^+^ response targeted against C10orf54 (T37M) using pHLA multimer staining assays (bottom right) (**b**). Lung CT scans from patient 13 with TNBC (top row) and longitudinal CD8^+^ response kinetics showing CD8^+^ T cells specific against GALNT6 (E579K) detected via pHLA multimer assay (middle row). Time-course analysis of neoantigen-specific CD8^+^ TCRs isolated from blood samples following the induction phase (as described in **a**, bottom row) (**c**). Lung CT scans from patient 38 with RCC (**d**). TCRseq, T cell receptor sequencing.

The CD8^+^ T cell responses persisted at a high level from the time of CR confirmation until the patient’s last follow-up while in CR at week 39. At 20 months after CR confirmation, the patient experienced disease progression, at which time only a fraction of autogene cevumeran-specific CD8^+^ TCRs (0.34%) and no autogene cevumeran-specific CD4^+^ TCRs were detectable in peripheral blood (Fig. 5a).

Patient 21, who had microsatellite stable, PD-L1-low rectal cancer after two lines of treatment with a 1.1 cm target lesion and a nontarget lesion in the lung, had a CR after treatment with 9 doses of 38 µg autogene cevumeran and atezolizumab with a duration of response of 8.2 months and remained on the study after >3 years as of the clinical data cutoff (40.7 months follow-up). Ex vivo ELISpot responses were detectable against 3 of their 20 neoantigens, of which the CD8^+^ T cell response directed against C10orf54 (T37M) was followed up and remained detectable up to 6.5 months after treatment start (Fig. 5b).

**Extended Data Fig. 8:**
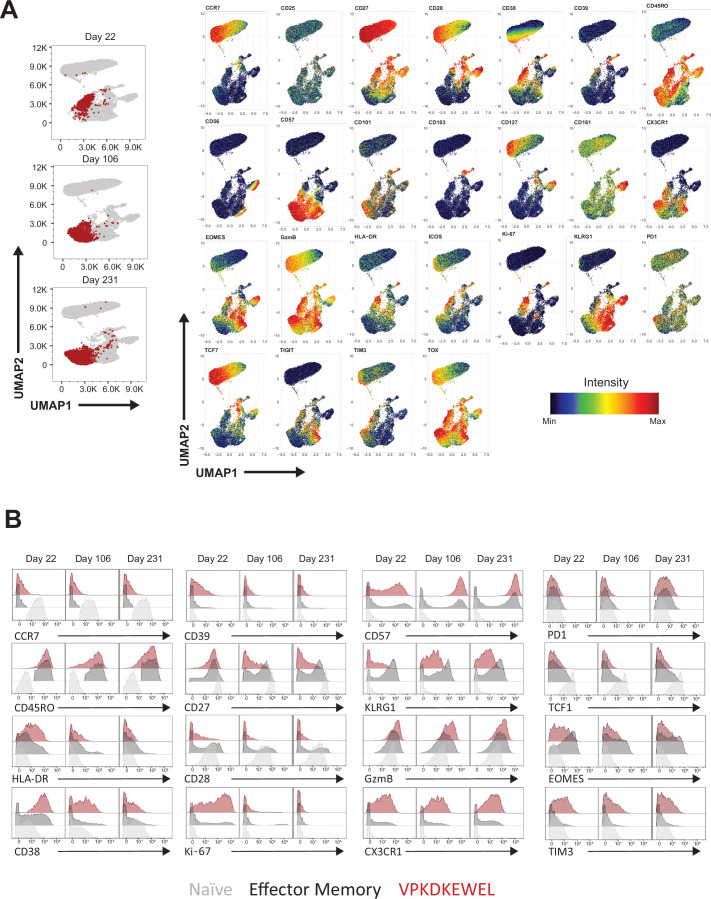
Phenotypic analysis of neoantigen-specific CD8 T cells from longitudinal time points using CYTOF in peripheral blood of patient 13. **a**, Frequencies of GALNT6 (E579K) -specific CD8 + T cells (VPKDKEWEL) in patient 13 at day 22, 106 and 232. **b**, UMAP (uniform manifold approximation and projection) of CD8 + T cells from patient 13 at day 22, 106 and 232. GALNT6 (E579K) -specific cells are highlighted. UMAP plots on the right display relative expression intensities of all phenotypic markers assessed. **c**, Histograms showing relative expression of individual markers on GALNT6 (E579K) -specific (red), Naïve (gray) or Effector memory (black) CD8 T cells from patient 13 at different times.

Patient 13 had PD-L1-high TNBC that progressed on a nivolumab-based investigational regimen. Upon treatment with 38 µg autogene cevumeran and atezolizumab, the patient experienced a confirmed PR including a reduction in the size of lung metastases with a duration of response of 9.9 months (29.9 months follow-up) (Fig. 5c). The patient mounted strong T cell responses to autogene cevumeran targets GALNT6 (E579K) and TTC13 (N144Y). About 9 months after treatment start, up to 11% of peripheral CD8^+^ T cells were represented by 13 CD8^+^ TCRs specific to those two neoantigens (Fig. 5c). Neoantigen-specific CD8^+^ T cells against GALNT6 (E579K) were detectable in peripheral blood after vaccination and were predominantly of the effector memory phenotype (Extended Data Fig. 3c). Deeper profiling of GALNT6 (E579K)-specific CD8 T cells by CyTOF revealed a proliferative and early activation signature marked by high Ki-67, HLA-DR, CD38 and ICOS and a transition to a more differentiated phenotype with a decline in CD27/CD28 and an increase in CD57 levels during the maintenance cycles. Throughout the course of analysis, the activation marker PD-1 remained stable, and these cells consistently showed high expression of the functional marker Granzyme B (Extended Data Fig. 8). These observations were consistent with phenotypes observed in neoantigen-specific CD8 T cells in other patients (Extended Data Fig. 4). The patient experienced progressive disease (PD) with progression of the nontarget lesion 11 months after initiation of vaccination and a decrease in neoantigen-specific TCRs in the periphery (Fig. 5c).

Patient 38 had kidney cancer and received four lines of systemic therapy, including nivolumab, before enrollment, and initially progressed while on study but subsequently responded with a PR (Extended Data Fig. 5). The patient developed T cell responses against 7 of 20 autogene cevumeran antigens in post-IVS ELISpot (Fig. 3c), including an effector memory phenotype ex vivo response (Extended Data Fig. 3c). The patient had pleural target lesions with the sum of the longest diameters (of baseline tumors) (SLD) at 108 mm, which was reduced to 10 mm after 13 months on treatment (Fig. 5d); 2.5% of tumor-infiltrating lymphocytes in the biopsy taken 5 months after start of treatment from a regressing pleural tumor lesion were autogene cevumeran-specific CD8^+^ T cells (Fig. 3h).

## Discussion

We present here a clinical and translational dataset of an individualized mRNA neoantigen cancer therapy, showing the feasibility of iNeST manufacturing at scale. Toxicities as monotherapy or in combination with atezolizumab included systemic reactions that were monitorable, manageable and reversible. Autogene cevumeran induced predominantly de novo CD4^+^ and CD8^+^ T cell responses against multiple neoantigens in the majority of patients, detectable up to 65 weeks following the last treatment of the longest followed-up patient with available data.

We identified a full set of 20 neoantigens in >80% of patients and the minimum number of 5 neoantigens in 98.5% of patients whose data entered the computational pipeline, showing that the iNeST approach is feasible across tumor types, including those traditionally viewed as having a low tumor mutational burden. This experience with autogene cevumeran shows the feasibility of on-demand manufacturing at scale for patients with locally advanced and metastasized cancers. This trial iteratively optimized the logistical, technical and operational aspects impacting the TATs to manufacturing processes used in the ongoing phase 2 studies of autogene cevumeran.

Autogene cevumeran monotherapy was generally well tolerated, with a safety profile characterized by transient, mild-to-moderate systemic reactions that were expected based on the desired innate immunostimulatory properties of nucleoside-unmodified uridine mRNA.

The systemic reactions were in line with the preclinically shown ability of RNA–LPX to engage TLR7/8 receptor-mediated inflammatory cytokine production^22^ and to elicit strong IL-1β secretion by myeloid cells^28^. A similar profile of proinflammatory cytokines was previously reported for a clinical trial using the same uridine RNA–LPX platform for a shared tumor-associated antigen vaccine^24^. The systemic reactions were typically self-limiting and occasionally required concomitant medication or dose reduction for management. The combination with atezolizumab did not result in new safety signals beyond the AE profile of either agent.

We used different orthogonal T cell assays to characterize the breadth, magnitude and kinetics of neoantigen-specific T cell responses in the blood. While technical challenges and a shortage of samples limited our ability to perform comprehensive peripheral blood mononuclear cell (PBMC)-based studies, we were able to study a representative number (94 of 213) of enrolled patients. With ex vivo ELISpot being used as the primary method of immune response analysis in 90 patients, our results with >70% immunogenicity probably underestimate the breadth of the autogene cevumeran-induced T cell response. In a small cohort of patients, post-IVS IFNγ-ELISpot identified twice as many autogene cevumeran neoantigens as immunogenic and a higher poly-epitopic diversity compared with ex vivo ELISpot. The magnitude of peripheral T cell responses against individual neoantigens varied. A substantial fraction of neoantigen-induced T cells were at levels below the high threshold of ex vivo detection by ELISpot, while by post-IVS ELISpot, pMHC multimer staining assays or TCR analysis, they were robustly detectable and persistent. Some patients had single-digit percentages of circulating CD8^+^ T cells directed against a single immunodominant autogene cevumeran neoantigen with or without additional weaker neoantigen responses. Other patients had T cell responses against a panel of multiple autogene cevumeran neoantigens, each of which was of low magnitude but added up to a considerable poly-epitopic autogene cevumeran-induced T cell count. Generally, the heterogeneity and individuality of the T cell immune response as an inherent feature of an individualized vaccine may make it difficult to define a distinct immune response correlate of mediating a clinical effect.

We detected preexisting T cell responses against only a small fraction of the neoantigen candidates identified by our computational pipeline for autogene cevumeran design. These responses were further amplified by autogene cevumeran. The vast majority of neoantigen autogene cevumeran-specific T cells were induced de novo, confirming that patients rarely mount T cells against tumor neoantigens spontaneously^8,29^ and indicating that autogene cevumeran in combination with atezolizumab contributed to priming or proper activation.

In addition to CD8^+^ T cells, autogene cevumeran induced de novo CD4^+^ T cells against a large fraction of neoantigens as intended by design^21^. Some cancer mutations gave rise to neoantigens that were immunogenic for both CD8^+^ and CD4^+^ T cells and were recognized in the context of several different restriction elements, showing that concurrent TCR- and phenotype-diverse immune responses can be mounted against a single cancer mutation. In subsets of patients, peptide-human leukocyte antigen multimers and TCR clonotype analysis revealed that responses against single individual neoantigens can exceed 5% of peripheral CD8^+^ T cells, reaching levels of circulating target-specific T cells comparable to those achieved with adoptive T cell therapy^30^. The highest cumulative TCR frequencies for autogene cevumeran-induced responses were above 20% of peripheral CD8^+^ T cells. Autogene cevumeran induced poly-epitopic neoantigen-specific T cell responses of high magnitude in a substantial number of patients, majority of which the majority of those tested trafficked into the tumor microenvironment.

This study was not primarily designed to investigate clinical efficacy. The study population represented a heterogenous group of patients with prognostically unfavorable features who were unlikely to respond to immunotherapy, having received multiple previous lines of immunosuppressive chemotherapy, and with PD-L1-low or PD-L-negative tumors. About one-quarter of patients had progressed before the peak of autogene cevumeran-induced T cell responses measured in peripheral blood was reached, approximately 6–9 weeks after treatment initiation in most patients. Although CPI-naive patients were enrolled in the dose expansion cohorts, to enable preliminary signal seeking with the autogene cevumeran–atezolizumab combination, the single-arm design was not suitable for capturing potential disease stabilization and survival benefit disproportionate to tumor regression. Interpretation of the cohorts in immunotherapy-responsive indications was limited by the small sample sizes and uncontrolled differences in baseline characteristics relative to historical CPI monotherapy benchmarks.

Despite these caveats, individual patients showed durable benefit associated with the induction of neoantigen-specific immune responses that were observed in a small subset of patients with different tumor types. CRs were seen with autogene cevumeran monotherapy for one patient with MSI-high gastric cancer and as a combination therapy for one patient with rectal cancer.

An ongoing proof-of-concept randomized phase 2 trial is evaluating whether autogene cevumeran plus CPI can improve clinical outcomes in previously untreated patients with advanced melanoma (NCT03815058). There is a strong rationale for applying T cell-inducing immune therapies in settings with low tumor load^31,32^. Study KEYNOTE-942 provides evidence that an mRNA-based individualized neoantigen therapy might be beneficial in the adjuvant setting^33^. Immune fitness is considered greater in the absence of widespread metastatic disease and previous extensive systemic immunosuppressive therapies^34,35^. Early cancers are also likely to have less clonal heterogeneity^36^ and be more readily eradicated by a relatively narrow immune response targeting a minority of autogene cevumeran-encoded neoantigens^32,37^. The tumor microenvironment of micrometastases may be less established and less exclusive of infiltrating immune cells than that of clinically apparent disseminated disease. Moreover, the tissue requirement and TAT for autogene cevumeran manufacturing is readily accommodated by standard clinical practice for patients undergoing curative-intent surgery followed by postoperative recovery. Accordingly, informed by the findings of this first-in-human trial, small studies in earlier settings have assessed autogene cevumeran. We reported preliminary results showing that post-(neo)adjuvant individualized RNA–LPX vaccination in early-stage TNBC elicits a strong and long-lasting polyneoepitope T cell response^38^. We further reported phase 1 data from 16 patients with resected PDAC who received autogene cevumeran and atezolizumab in addition to standard chemotherapy, showing a promising correlation between neoantigen-specific immune response and prolonged recurrence-free survival^25,26^. A randomized phase 2 study comparing standard-of-care chemotherapy with or without autogene cevumeran and atezolizumab in patients with resected PDAC (NCT05968326) is underway to confirm these findings. In addition, a randomized phase 2 study of autogene cevumeran versus best supportive care in patients with CRC who are ctDNA+ after resection and have completed standard adjuvant chemotherapy (NCT04486378) is ongoing.

In conclusion, we show the feasibility and tolerability of autogene cevumeran, an individualized neoantigen-specific mRNA therapy, in patients with advanced solid tumors. The immunogenicity of autogene cevumeran compares favorably with alternative vaccine platforms^14,16^. Observations from this trial and companion efforts support the continued development of autogene cevumeran in earlier lines of therapy in which the mechanism of action is likely to have the greatest impact on patient outcomes. These data will also inform iterative improvements in the neoantigen prediction algorithm and platform, and exploration of rational therapeutic combinations.

## Methods

### Study overview

GO39733 is a phase 1 open-label study of autogene cevumeran as monotherapy (phase 1a) or in combination with 1,200 mg atezolizumab (phase 1b) in patients with locally advanced or metastatic solid tumors (NCT03289962). A total of 213 patients were enrolled across 33 sites in North America and Europe between 21 December 2017 and 12 January 2022. Data shown are up to the clinical cutoff date of 14 January 2022.

### Inclusion and ethics

The study was conducted in accordance with the Declaration of Helsinki, International Conference on Harmonization E6 guidelines and Good Clinical Practice guidelines. The protocol was approved by the institutional review boards at Karolinska Hospital, UZ Gent, Southampton General Hospital, Stanford Cancer Center, Universitatsmedizin der Johannes Gutenberg Universitat Mainz, Universitaetsklinikum Essen, Massachusetts General Hospital, Sarah Cannon Research Institute—Tennessee Oncology, Hospital Univ Vall d’Hebron Servicio de Oncologia, Columbia University Medical Center, Sint Augustinus Wilrijk, Nationales Centrum fur Tumorerkrankungen Heidelberg, Dana-Farber Cancer Institute, Seattle Cancer Care Alliance, CHU Sart Tilman, Akademiska sjukhuset, Onkologkliniken, UCSF Comprehensive Cancer Center, Antoni van Leeuwenhoek Ziekenhuis, Providence Oncology and Hematology Care Eastside, Fachklinik fur Lungenerkrankungen, The Ottawa Hospital Cancer Centre, Universitair Medisch Centrum Utrecht, University of Oklahoma Health Sciences Center, Clinica Universitaria de Navarra, LungenClinic Groshansdorf, Georgetown University, Beth Israel Deaconess Medical Center, Leiden University Medical Center, University of Pittsburgh Medical Center and Klinisch-Pharmakologisches Studienzentrum. Patient consent was obtained before enrollment. Patients were not compensated for their participation in the study.

### Study objectives

The primary objective was safety and tolerability of autogene cevumeran as monotherapy and in combination with atezolizumab. Exploratory objectives included pharmacokinetics and pharmacodynamics of autogene cevumeran, preliminary antitumor activity and immunogenicity.

### Study design and treatment

In phase 1a, patients were enrolled in dose escalation. In phase 1b, patients were enrolled in dose escalation, CPI-naive and CPI-experienced expansion cohorts; a serial biopsy cohort; or a biomarker substudy. Crossover from phase 1a to phase 1b after disease progression or stable disease at cycle 7 was allowed.

Doses of autogene cevumeran were escalated in a standard 3 + 3 design ranging from 25 μg to 100 μg in phase 1a and 25 μg to 50 μg in phase 1b. Due to limited dose escalation steps, the standard 3 + 3 design was adopted for its safety and simplicity on both concept and operation. In the phase 1b dose expansion, patients received 15 μg or 25 μg autogene cevumeran with atezolizumab.

The treatment regimen for both phases included an induction stage of eight doses of autogene cevumeran administered intravenously over the first 9 weeks followed by periodic booster doses during the maintenance stage until disease progression. Makeup doses of autogene cevumeran were allowed. In phase 1b, atezolizumab was administered intravenously on day 1 of each 21 day cycle until disease progression. Patients fulfilling prespecified criteria were eligible to continue study treatment beyond disease progression. Patients in the serial biopsy cohort and CPI-naive patients for whom manufacturing of the autogene cevumeran was delayed received an initial dose of atezolizumab (cycle 1, day 1) and started the induction stage with autogene cevumeran (cycle 2, day 1).

### Patients

Eligible patients had locally advanced, recurrent or metastatic incurable malignancy that had progressed after at least one available standard therapy, for whom standard therapy does not exist, has proven to be ineffective or intolerable, or is considered inappropriate; for whom a clinical trial of an investigational agent is a recognized standard of care; or for whom a clinical trial of an investigational agent in combination with an anti-PD-L1 antibody is considered an acceptable treatment option (phase 1b only).

Enrollment in the phase 1a dose escalation was restricted to the following tumor types: gastric or gastroesophageal junction, esophageal, sarcoma, prostate, uterine or endometrial, breast (HER2+ and/or HR+), and ovarian and thyroid. Enrollment in the phase 1b dose escalation, serial biopsy cohort and biomarker substudy was restricted to the following tumor types: NSCLC, small cell lung cancer, UC, CRC, RCC, TNBC, head and neck squamous cell carcinoma, melanoma, cervical, anal, Merkel cell carcinoma, squamous cell carcinoma of the skin, hepatocellular carcinoma (non-viral) and MSI-high tumors.

Patients were ≥18 years old with an Eastern Cooperative Oncology Group performance status of 0–1 and were required to have ≥5 identified neoantigens to be eligible.

### Safety

Safety was assessed through summaries of DLTs, AEs, changes in laboratory test results, changes in vital signs and ECGs, and exposure to study treatment. AEs were reported per National Cancer Institute Common Terminology Criteria for Adverse Events (CTCAE), v.5.0, for 90 days after the last dose of autogene cevumeran and/or atezolizumab or before the initiation of a new systemic anticancer treatment. An Internal Monitoring Committee reviewed safety at regular intervals.

Potential immune-mediated AEs were identified based on a predefined set of AE group terms commonly associated with defined immune-mediated AEs, the temporal relationship to receiving study drug and considering use of systemic corticosteroid treatment at the time of AE.

### Efficacy

Response was assessed by the investigator based on physical examinations and imaging modalities using Response Evaluation Criteria in Solid Tumours v.1.1 (RECIST v.1.1). The sponsor (Genentech) derived the BOR and confirmed the BOR per RECIST v.1.1 based on entries for all target lesions, nontarget lesions and new lesions up to the clinical cutoff date.

### Manufacture of autogene cevumeran

The drug product was manufactured, analyzed and released as described previously^24^. Patients signed a prescreening informed consent form to allow the collection and testing of archival or fresh tumor specimens, before signing the main study informed consent form. Biopsies with a tumor content as low as 10% sufficed for the manufacturing of autogene cevumeran. The manufacturing process is described briefly below.

### Next-generation sequencing

Tumor DNA and RNA were extracted from formalin-fixed paraffin-embedded (FFPE) tumor tissue using a modified QIAamp DNA FFPE Tissue Kit (QIAGEN) or ExpressArt Clear FFPE RNAready kit (AmpTec). DNA was extracted from blood-derived PBMCs using a QIAGEN Dneasy Blood and Tissue Kit. Targeted RNA-seq libraries were constructed in duplicate from 100 ng FFPE tumor RNA using the NEBNext RNA First Strand Synthesis Module, the NEBNext Ultra Directional RNA Second Strand Synthesis Module and a modified Agilent SureSelect XT V6 Human All Exon kit. DNA whole-exome libraries were constructed in duplicate from 100 ng of FFPE tumor DNA and matching PBMC DNA using a modified Agilent SureSelect XT V6 Human All Exon kit. Libraries were clustered at 10 pM using the Illumina HiSeq 4000 PE Cluster Kit. All libraries were sequenced paired-end 50 nt on an Illumina HiSeq 4000 platform.

### Bioinformatics and mutation discovery

Genomics-related data analysis was coordinated by a Python software pipeline. DNA reads were aligned to the reference genome hg19 with bwa (v.0.7.10). Alignment files were converted to the BAM format using samtools (v.0.1.19). Somatic single-nucleotide variants and short indels were called by comparing aligned tumor and PBMC DNA reads.

Genomic coordinates of identified variants were compared with UCSC Known Genes transcript coordinates. Synonymous and nonsense mutations were filtered. For variants changing the amino acid sequence, massively parallel sequencing (MPS) of targets was defined as the sequence 13 amino acids N-terminal and 13 C-terminal of the changed amino acid(s). For frameshift indels, the sequence from the changed amino acid until the next stop codon was considered. Germline variants in the region of mutated peptides were identified using samtools based on the matching PBMC DNA. Protein-changing germline variants were first phased based on the RNA-seq reads and, if in phase with somatic mutations, were included in the patient-specific neoantigen autogene cevumeran target.

RNA reads were aligned to the hg19 transcriptome using sailfish (v.0.7.6). Non-expressed transcripts were filtered. RNA reads were aligned to the hg19 reference genome using STAR (v.2.4.2a).

### Neoantigen prioritization and selection

HLA binding affinity was predicted with T cell prediction tools (IEDB v.2.13), using the IEDB-recommended mode for affinity to HLA class I (HLA-A, HLA-B and HLA-C) and the consensus3 method for affinity to HLA class II (HLA-DRB). Of all predictions for a single variant, the best consensus score was associated with the respective MPS.

Up to 46 MPS from all identified MPS were prioritized by a custom Python script. First, only somatic mutations with a variant allele frequency in RNA > 0 were considered. From these, ≤5 MPS from indels were selected based on their HLA class I binding affinity, ≤20 MPS from single nucleotide variants (SNVs) based on their HLA class I binding affinity and transcript expression ≥10 RPKM, ≤20 MPS from SNVs based on their HLA class I binding affinity and transcript expression ≥1 RPKM, and further MPS from SNVs based on their transcript expression to reach 46 MPS in total. When fewer than 46 MPS could be selected, somatic mutations with a variant allele frequency in RNA of zero were added based on the HLA class I score and transcript expression.

An algorithm in R was used to select ≤20 neoantigen autogene cevumeran targets from the list of prioritized MPS based on HLA I and HLA II binding predictions, transcript expression, variant allele frequency and other criteria. The selection was reviewed by a review board.

### Good Manufacturing Practice manufacturing of RNA–LPX

Two synthetic DNA fragments, each coding ≤10 neoantigen autogene cevumeran targets (SNVs and short insertions and deletions) connected by 30 bp non-immunogenic glycine and serine linkers, were cloned into a starting vector^18^ containing the secretory signal peptide (SEC)^21^ (MRVMAPRTLILLLSGALALTETWAGS) and the MHC class I trafficking domain (MITD) sequences^21^ (IVGIVAGLAVLAVVVIGAVVATVMCRRKSSGGKGGSYSQAASSDSAQGSDVSLTA) for fusion to mutated sequence concatamers for optimized routing to HLA class I and II pathways. The starting vectors also contained 3' UTR^20^ (LVLHARNASCPFPVLGTPSLPRPRVPGMLPPPPAPLTTSASSRHL) and backbone sequence elements for improved RNA stability and translational efficiency. Linearized DNA fragments were produced from pDNA via PCR, spectrophotometrically quantified, identified with Sanger sequencing and in vitro transcribed T7 RNA polymerase as previously described^17^ in the presence of ATP, CTP, UTP, GTP and β-S-ARCA(D1) cap analog in a cleanroom environment. RNA was purified using magnetic particles, and integrity was assessed by gel electrophoresis and microfluidic capillary electrophoresis; concentration, pH, osmolality, potency and endotoxin level were measured.

Net cationic charge liposomes, manufactured using an adopted proprietary protocol^39,40^, were mixed with RNAs to form RNA–LPXs. Release analysis for the RNA–LPXs included determination of appearance, particle size and polydispersity, subvisible particles, RNA integrity, RNA content, pH, osmolality, endotoxin testing and sterility. For administration, the thawed product was diluted with 0.9% aqueous NaCl.

### Pharmacodynamics

Pharmacodynamic biomarkers were assessed from 94 patients with adequate tumor tissue and/or blood for analysis.

### Immune response assessment

Immune response induced by autogene cevumeran was assessed by ex vivo IFNγ-ELISpot in 90 patients who had reached cycle 4 (Fig. 1a) with sufficient material available. This was performed as previously described^25^ using samples collected at baseline and after eight or seven vaccinations for majority of the patients (76 and 11, respectively); two and one patient’s samples were analyzed after six and five doses, respectively. The timing of phase 1a crossover to phase 1b and the timing of the start of atezolizumab treatment in the biomarker substudy were considered in the analysis. Synthetic 15-mer peptides with 11 amino acid overlaps covering the neoantigen sequences (overlapping peptide pools) or 8–11-mer peptides were used in immunogenicity assessments. All synthetic peptides were purchased from JPT Peptide Technologies and dissolved in 10% DMSO to a final concentration of 3 mM.

An IFNγ ELISpot assay following the IVS of CD4^+^ and CD8^+^ T cells was performed in patients from the biomarker substudy who provided leukapheresis, as described previously^24^ with the following exception: in vitro expansion was performed with OLPs for 11 days. ELISpot plates were scanned with the CTL S6CORE analyzer (ImmunoCapture Image Acquisition Software v.6.6) and analyzed by with ImmunoSpot Professional Software v.5.4. For the definition of a positive response in each sample, peptide-stimulated spot counts were compared with those of control peptide-loaded target cells using an in-house statistical analysis tool. T cell autogene cevumeran responses were defined with an increase in spot count after vaccination of at least twofold.

### Multiplexed multimer staining

HLA–peptide binding affinities were predicted across all pairs of HLA alleles and mutation-containing 8–11-mer peptides using NetMHCpan. For each neoantigen, a list of HLA–peptide pairs with a percentile rank score of <5% across all pairs was generated. The top three lowest percentile rank (the highest binding likelihood) combinations were selected for generating tetramerized pHLA–complexes. A two-color combinatorial tetramer staining approach was used. PBMCs were first stained with respective pHLA multimers and then with an antibody mix consisting of CD8^+^ and dump channel antibodies and a dead cell marker. The following antibodies (clones) were used: CD3 (UCHT1), CD14 (MφP9), CD8 (RPA-T8) and CD45RO (UCHL1) (BD Biosciences); CD16 (3G8), CD19 (SJ25C1), CD4 (RPA-T4), PD-1 (EH12.2H7) and CCR7 (G043H7) (Biolegend); and Live/Dead Fixable Dead Cell Staining Kit (Thermo Fisher Scientific). Tetramer-specific CD8^+^ T cells were analyzed as single, live lymphocytes, CD4^−^CD14^−^CD16^−^CD19^−^CD3^+^CD8^+^ T cells. Analytical processing includes Boolean deconvolution steps to eliminate single- and triple-stained tetramer-positive events. The gating strategy is provided in Supplementary Fig. 1.

### Mass cytometry

For high-dimensional phenotyping, ten patients with measurable CD8^+^ T cells were selected (Supplementary Table 9); longitudinal analysis was performed by ImmunoScape, as described previously^38,39^. PBMC availability during the induction and maintenance phases from different patients is outlined in Supplementary Table 9. Briefly, PBMCs from patient samples were sorted for live lymphocytes and stained with heavy-metal-labeled pHLA tetramer cocktails in two different coding configurations containing all patient-specific neoepitope candidates and 33 different metal-labeled antibodies for immune cell subset discrimination and phenotypic profiling (Supplementary Table 9). Longitudinal patient samples and staining configurations were individually barcoded and acquired together on a HELIOS mass cytometer (Fluidigm). Bona fide tetramer-positive events were evaluated using a scoring system based on different criteria, and CD8^+^ T cells were phenotypically profiled using high-dimensional analysis methods and assessed for treatment-associated phenotypic changes as described previously^41,42^. All samples were run in technical replicates using a second configuration staining with a completely different barcoding scheme. Each patient sample was manually de-barcoded (time-point and tetramer staining configuration) followed by gating on live CD8^+^ and CD4^+^ T cells (CD45^+^DNA^+^cisplatin^–^CD3^+^ cells) after gating out B cells (CD19^+^), monocytes (CD14^+^) and gamma–delta T cells using FlowJo (Tree Star). High-dimensional and statistical data analysis and visualization were performed using ImmunoScape’s cloud-based analytical software Cytographer, FlowJo and GraphPad Prism software. Phenotypic profiles and sample distributions were shown using principal component analysis and uniform manifold approximation and projection for high-dimensionality reduction.

### Bulk TCR sequencing analysis

Total RNA was extracted from 3–5 × 10^5^ magnetic-activated cell-sorted CD8^+^ or CD4^+^ T cells or fresh frozen needle tumor biopsies using the RNeasy Mini kit (QIAGEN). Total RNA was eluted in 30 μl water and used for bulk TCR sequencing. Bulk TCR profiling libraries were generated using the SMARTer Human TCR a/b Profiling kit from Clontech Laboratories. Library sequencing was performed on an Illumina MiSeq sequencer using the 600-cycle MiSeq Reagent Kit v3 with paired-end, 2 × 300-bp reads. The data generated on the MiSeq Sequencer were demultiplexed using the Bcl2Fastq software (Illumina). Fasta sequences were edited using the trimmomatic v.0.36 software and analyzed with the MiXCR software.

### Single-cell TCR sequencing analysis

Twenty thousand CD8^+^ or CD4^+^ magnetic-activated cell-sorted T cells were used for variable diversity joining analysis (see user guide for Chromium Next GEM Single Cell V(d)J Reagent Kits v.1.1, 10x Genomics, CG000207 Rev D). TCR library sequencing was performed on an Illumina MiSeq sequencer using the MiSeq Reagent Kit, 600 cycles, v.3, with paired-end, 2 × 150-bp reads. Raw sequencing data were processed using the Cell Ranger software (10x Genomics).

### TCR candidate selection and functional characterization

The most enriched paired TCR chains were identified from the single-cell and bulk TCR sequencing analysis ex vivo. TCR V(D)J genes of the selected candidates were synthesized by Twist Bioscience, then cloned into vector backbones encoding the TCR constant region domains. A PCR-amplified, full-length linear TCR chain template was used to produce the in vitro transcription (IVT)-RNA for the in vitro assays performed for TCR validation. The functionality of the selected TCR candidates was tested using a Jurkat NFAT-reporter cell line. After electroporation of TCR alpha and beta encoding IVT-RNA and a 20-h incubation period, 2 × 10^4^ Jurkat cells were co-cultured with K562 cells or autologous CD14^+^ monocytes at a 5:1 ratio in a 384-well plate with 25 μl of medium (RPMI1640 + 10% non-heat-inactivated FBS) per well. Before the co-culture, the antigen-presenting cells were transfected with IVT-RNA encoding the patients’ HLA alleles (K562 only) and loaded with neoantigen target peptide pools or transfected with RNA encoding for autogene cevumeran targets. After 6 h, an equal volume (25 μl) of luciferin (Bio-Glo, Promega) was added to each well and the luciferase activity was measured using a luminescence plate reader. The measured luminescence signal corresponded to the level of TCR-mediated activation in the Jurkat cells. For each TCR, the fold change of luminescence compared with the ‘effectors-only control’ was calculated and a cutoff of a twofold change was used to determine specific TCRs. Neoantigen-specific CD4^+^ TCRs for patient 31 were selected analyzing the enrichment post-IVS. Neoantigen-specific CD4^+^ TCRs for patient 85 were isolated by sorting the activated CD4^+^CD154^+^CD69^+^ T cells after antigen stimulation (6 h).

### Cytokines

Plasma samples were collected before and 4–6 h after autogene cevumeran infusion and stored at −80 °C. Cytokine levels were measured using a human pan-IFNα ELISA (PBL Assay Science), Simple Plex (R&D systems) for IL-6 and TNF, and Simoa (Quanterix) for IFNγ, IL-1b and IL-12p70.

### PD-L1 immunohistochemistry

Pretreatment FFPE tumor tissue was stained for PD-L1 using a proprietary diagnostic anti-human PD-L1 monoclonal antibody (SP142)^43^. Samples were scored for PD-L1 expression on tumor-infiltrating ICs—macrophages, DCs and lymphocytes—or on TCs. PD-L1-low or PD-L1-negative disease was identified as <5% IC and TC with the SP142 assay.

### Statistical analyses

This study planned to enroll 307–770 patients, to investigate safety, pharmacodynamics and clinical activity. No explicit power and type 1 error considerations were made, and all analyses were descriptive and exploratory. All patients who received at least one dose of autogene cevumeran or atezolizumab were included in the safety and activity analyses. Data from patients enrolled into an adjuvant NSCLC substudy or into cohorts evaluating prophylactic corticosteroids with autogene cevumeran administration were not included in this paper (*n* = 58).

All patients with available PFS data were included in the analysis of the impact of selected baseline factors on PFS. For patients who crossed over from phase 1a to 1b, only phase 1a PFS data were included. Analysis of some factors was performed with a reduced number of patients owing to the limitation of underlying baseline factor data sets. Cox proportional hazard analysis was performed according to a basic univariate model: *h*(*t*) = *h*_0_(*t*) × exp(*b*_1_*x*_1_). For a better comparison of hazard ratios among diverse baseline factors, all feature values were dichotomized in respective high and low subgroups along the median of the respective factor and of the analyzed population.

### Reporting summary

Further information on research design is available in the Nature Portfolio Reporting Summary linked to this article.

## Online content

Any methods, additional references, Nature Portfolio reporting summaries, source data, extended data, supplementary information, acknowledgements, peer review information; details of author contributions and competing interests; and statements of data and code availability are available at 10.1038/s41591-024-03334-7.

## Supplementary information

Supplementary Information Supplementary Tables 1–9 and Fig. 1.

Reporting Summary

## Figures and Tables

**Extended Data Table 1:**
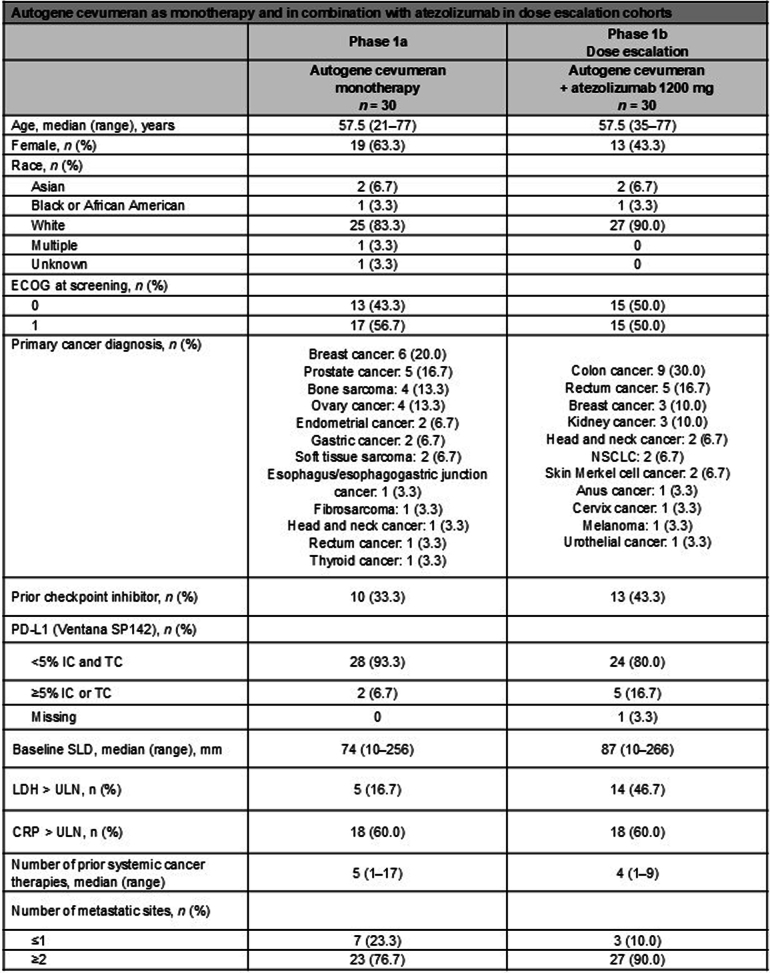
Patient baseline characteristics of autogene cevumeran as monotherapy (phase 1a) and in combination with atezolizumab in dose escalation cohorts (phase 1b)

PD-L1 positivity was assayed by immunocytochemistry using the Ventana SP142 test with a cut-off of <5% PD-L1 positivity of immune cells and tumor cells. SLD, sum of longest diameters (of baseline tumors).

**Extended Data Table 2:**
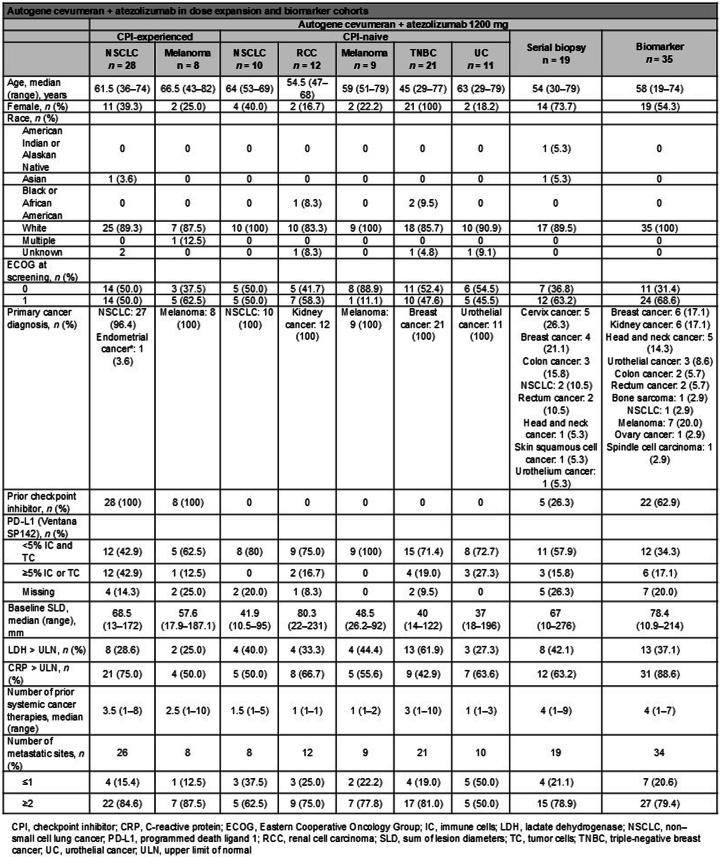
Patient baseline characteristics of autogene cevumeran in combination with atezolizumab (phase 1b) in dose expansion and biomarker cohorts

PD-L1 positivity was assayed by immunocytochemistry using the Ventana SP142 test with a cut-off of <5% PD-L1 positivity of immune cells and tumor cells.

* Patient was initially diagnosed with NSCLC but was later determined to have endometrial cancer and subsequently discontinued from study. SLD, sum of longest diameters (of baseline tumors).

## Data Availability

For eligible studies, qualified researchers may request access to individual patient-level clinical data through a data request platform. At the time of writing, this request platform is Vivli: https://vivli.org/ourmember/roche/. As this study is ongoing, access to patient-level data from this trial will not be available until at least 18 months after the last patient visit and a clinical study report has been completed. After that time, requests for data will be assessed by an independent review panel, which decides whether the data will be provided. On average, it takes a few months to access data in the Vivli platform, but the timeline will vary depending on the number of data contributors, the number of studies and your availability to respond to comments. Once approved, the data are available for up to 24 months. For up-to-date details on Roche’s Global Policy on the Sharing of Clinical Information and how to request access to related clinical study documents, see https://www.roche.com/innovation/process/clinical-trials/data-sharing. Anonymized records for individual patients across more than one data source external to Roche cannot, and should not, be linked owing to a potential increase in risk of patient reidentification.

## References

[1] Sharma, P. et al. Immune checkpoint therapy—current perspectives and future directions. *Cell* **186**, 1652–1669 (2023). 37059068 10.1016/j.cell.2023.03.006

[2] Herbst, R. S. et al. Predictive correlates of response to the anti-PD-L1 antibody MPDL3280A in cancer patients. *Nature* **515**, 563–567 (2014). 25428504 10.1038/nature14011 PMC4836193

[3] Rizvi, N. A. et al. Cancer immunology. Mutational landscape determines sensitivity to PD-1 blockade in non-small cell lung cancer. *Science* **348**, 124–128 (2015). 25765070 10.1126/science.aaa1348 PMC4993154

[4] Samstein, R. M. et al. Tumor mutational load predicts survival after immunotherapy across multiple cancer types. *Nat. Genet.* **51**, 202–206 (2019). 30643254 10.1038/s41588-018-0312-8 PMC6365097

[5] Snyder, A. et al. Genetic basis for clinical response to CTLA-4 blockade in melanoma. *N. Engl. J. Med.* **371**, 2189–2199 (2014). 25409260 10.1056/NEJMoa1406498 PMC4315319

[6] Van Allen, E. M. et al. Genomic correlates of response to CTLA-4 blockade in metastatic melanoma. *Science* **350**, 207–211 (2015). 26359337 10.1126/science.aad0095 PMC5054517

[7] Parkhurst, M. R. et al. Unique neoantigens arise from somatic mutations in patients with gastrointestinal cancers. *Cancer Discov.* **9**, 1022–1035 (2019). 31164343 10.1158/2159-8290.CD-18-1494 PMC7138461

[8] Tran, E. et al. Immunogenicity of somatic mutations in human gastrointestinal cancers. *Science* **350**, 1387–1390 (2015). 26516200 10.1126/science.aad1253 PMC7445892

[9] Rappaport, A. R. et al. A shared neoantigen vaccine combined with immune checkpoint blockade for advanced metastatic solid tumors: phase 1 trial interim results. *Nat. Med.* **30**, 1013–1022 (2024). 38538867 10.1038/s41591-024-02851-9

[10] Yarchoan, M. et al. Personalized neoantigen vaccine and pembrolizumab in advanced hepatocellular carcinoma: a phase 1/2 trial. *Nat. Med.* **30**, 1044–1053 (2024). 38584166 10.1038/s41591-024-02894-y PMC11031401

[11] Tureci, O. et al. Targeting the heterogeneity of cancer with individualized neoepitope vaccines. *Clin. Cancer Res.* **22**, 1885–1896 (2016). 27084742 10.1158/1078-0432.CCR-15-1509

[12] Vormehr, M. et al. Mutanome directed cancer immunotherapy. *Curr. Opin. Immunol.* **39**, 14–22 (2016). 26716729 10.1016/j.coi.2015.12.001

[13] Carreno, B. M. et al. Cancer immunotherapy. A dendritic cell vaccine increases the breadth and diversity of melanoma neoantigen-specific T cells. *Science* **348**, 803–808 (2015). 25837513 10.1126/science.aaa3828 PMC4549796

[14] Hilf, N. et al. Actively personalized vaccination trial for newly diagnosed glioblastoma. *Nature* **565**, 240–245 (2019). 30568303 10.1038/s41586-018-0810-y

[15] Keskin, D. B. et al. Neoantigen vaccine generates intratumoral T cell responses in phase Ib glioblastoma trial. *Nature* **565**, 234–239 (2019). 30568305 10.1038/s41586-018-0792-9 PMC6546179

[16] Ott, P. A. et al. An immunogenic personal neoantigen vaccine for patients with melanoma. *Nature* **547**, 217–221 (2017). 28678778 10.1038/nature22991 PMC5577644

[17] Sahin, U. et al. Personalized RNA mutanome vaccines mobilize poly-specific therapeutic immunity against cancer. *Nature* **547**, 222–226 (2017). 28678784 10.1038/nature23003

[18] Holtkamp, S. et al. Modification of antigen-encoding RNA increases stability, translational efficacy, and T-cell stimulatory capacity of dendritic cells. *Blood* **108**, 4009–4017 (2006). 16940422 10.1182/blood-2006-04-015024

[19] Kuhn, A. N. et al. Determinants of intracellular RNA pharmacokinetics: implications for RNA-based immunotherapeutics. *RNA Biol.* **8**, 35–43 (2011). 21289486 10.4161/rna.8.1.13767

[20] Orlandini von Niessen, A. G. et al. Improving mRNA-based therapeutic gene delivery by expression-augmenting 3′ UTRs identified by cellular library screening. *Mol. Ther.* **27**, 824–836 (2019). 30638957 10.1016/j.ymthe.2018.12.011 PMC6453560

[21] Kreiter, S. et al. Increased antigen presentation efficiency by coupling antigens to MHC class I trafficking signals. *J. Immunol.* **180**, 309–318 (2008). 18097032 10.4049/jimmunol.180.1.309

[22] Kranz, L. M. et al. Systemic RNA delivery to dendritic cells exploits antiviral defence for cancer immunotherapy. *Nature* **534**, 396–401 (2016). 27281205 10.1038/nature18300

[23] De Vries, J. & Figdor, C. Immunotherapy: cancer vaccine triggers antiviral-type defences. *Nature* **534**, 329–331 (2016). 27281206 10.1038/nature18443

[24] Sahin, U. et al. An RNA vaccine drives immunity in checkpoint-inhibitor-treated melanoma. *Nature* **585**, 107–112 (2020). 32728218 10.1038/s41586-020-2537-9

[25] Rojas, L. A. et al. Personalized RNA neoantigen vaccines stimulate T cells in pancreatic cancer. *Nature* **618**, 144–150 (2023). 37165196 10.1038/s41586-023-06063-y PMC10171177

[26] Guasp, P. et al. Personalized RNA neoantigen vaccines induce long-lived CD8^+^ T effector cells in pancreatic cancer. *Cancer Immunol. Res.* **12**, abstract PR-06 (2024).

[27] Genentech. TECENTRIQ® (atezolizumab injection) (package insert). https://www.gene.com/download/pdf/tecentriq_prescribing.pdf (2024).

[28] Tahtinen, S. et al. IL-1 and IL-1ra are key regulators of the inflammatory response to RNA vaccines. *Nat. Immunol.* **23**, 532–542 (2022). 35332327 10.1038/s41590-022-01160-y

[29] Kristensen, N. P. et al. Neoantigen-reactive CD8^+^ T cells affect clinical outcome of adoptive cell therapy with tumor-infiltrating lymphocytes in melanoma. *J. Clin. Invest.* **132**, e150535 (2022). 34813506 10.1172/JCI150535 PMC8759789

[30] Mackensen, A. et al. Phase I study of adoptive T-cell therapy using antigen-specific CD8^+^ T cells for the treatment of patients with metastatic melanoma. *J. Clin. Oncol.* **24**, 5060–5069 (2006). 17075125 10.1200/JCO.2006.07.1100

[31] Liu, J. et al. Cancer vaccines as promising immuno-therapeutics: platforms and current progress. *J. Hematol. Oncol.* **15**, 28 (2022). 35303904 10.1186/s13045-022-01247-x PMC8931585

[32] Saleh, R. & Elkord, E. Acquired resistance to cancer immunotherapy: role of tumor-mediated immunosuppression. *Semin. Cancer Biol.* **65**, 13–27 (2020). 31362073 10.1016/j.semcancer.2019.07.017

[33] Weber, J. S. et al. Individualised neoantigen therapy mRNA-4157 (V940) plus pembrolizumab versus pembrolizumab monotherapy in resected melanoma (KEYNOTE-942): a randomised, phase 2b study. *Lancet* **403**, 632–644 (2024). 38246194 10.1016/S0140-6736(23)02268-7

[34] Mackall, C. L. et al. Lymphocyte depletion during treatment with intensive chemotherapy for cancer. *Blood* **84**, 2221–2228 (1994). 7919339

[35] Mathios, D. et al. Anti-PD-1 antitumor immunity is enhanced by local and abrogated by systemic chemotherapy in GBM. *Sci. Transl. Med.* **8**, 370ra180 (2016). 28003545 10.1126/scitranslmed.aag2942 PMC5724383

[36] Mroz, E. A. & Rocco, J. W. MATH, a novel measure of intratumor genetic heterogeneity, is high in poor-outcome classes of head and neck squamous cell carcinoma. *Oral Oncol.* **49**, 211–215 (2013). 23079694 10.1016/j.oraloncology.2012.09.007 PMC3570658

[37] Jhunjhunwala, S., Hammer, C. & Delamarre, L. Antigen presentation in cancer: insights into tumour immunogenicity and immune evasion. *Nat. Rev. Cancer* **21**, 298–312 (2021). 33750922 10.1038/s41568-021-00339-z

[38] Schmidt, M. et al. T-cell responses induced by an individualized neoantigen specific immune therapy in post (neo)adjuvant patients with triple negative breast cancer. *Ann. Oncol.* **31**, S276 (2020).

[39] Grabbe, S. et al. Translating nanoparticulate-personalized cancer vaccines into clinical applications: case study with RNA-lipoplexes for the treatment of melanoma. *Nanomedicine* **11**, 2723–2734 (2016). 27700619 10.2217/nnm-2016-0275

[40] Batzri, S. & Korn, E. D. Single bilayer liposomes prepared without sonication. *Biochim. Biophys. Acta* **298**, 1015–1019 (1973). 4738145 10.1016/0005-2736(73)90408-2

[41] Fehlings, M. et al. Late-differentiated effector neoantigen-specific CD8+ T cells are enriched in peripheral blood of non-small cell lung carcinoma patients responding to atezolizumab treatment. *J. Immunother. Cancer* **7**, 249 (2019). 31511069 10.1186/s40425-019-0695-9 PMC6740011

[42] Fehlings, M. et al. Single-cell analysis reveals clonally expanded tumor-associated CD57^+^ CD8 T cells are enriched in the periphery of patients with metastatic urothelial cancer responding to PD-L1 blockade. *J. Immunother. Cancer* **10**, e004759 (2022). 35981786 10.1136/jitc-2022-004759 PMC9394212

[43] Vennapusa, B. et al. Development of a PD-L1 complementary diagnostic immunohistochemistry assay (SP142) for atezolizumab. *Appl Immunohistochem. Mol. Morphol.* **27**, 92–100 (2019). 29346180 10.1097/PAI.0000000000000594 PMC6369970

