# Supplementary material for: Autogene cevumeran with or without atezolizumab in advanced solid tumors: a phase 1 trial
Source: Nat Med. 2025 Jan 6;31(1):152–64. doi: 10.1038/s41591-024-03334-7 (PMC11750724; doi:10.1038/s41591-024-03334-7)

nature medicine

Supplementary information https://doi.org/10.1038/s41591-024-03334-7

Autogene cevumeran with or without atezolizumab in advanced solid tumors: a phase 1 trial

In the format provided by the authors and unedited

SUPPLEMENTARY MATERIAL

Table of Contents

Supplementary Table 1. Exposure and disposition of patients treated with autogene cevumeran monotherapy (phase 1a) and with atezolizumab (phase 1b)

Supplementary Table 2. Treatment-related adverse events and individual signs and symptoms of systemic reactions in ≥5% of phase 1a and b patients

Supplementary Table 3. Incidence of immune-mediated adverse events observed in phase 1a and phase 1b

Supplementary Table 4. Sampling for immune monitoring by cohort

Supplementary Table 5. List of neoantigen specific TCRs

Supplementary Table 6. Clinical activity in patients treated with autogene cevumeran monotherapy and with atezolizumab

Supplementary Table 7. Clinical activity in patients treated with autogene cevumeran with atezolizumab in phase 1b dose expansion

Supplementary Table 8. Clinical activity in patients treated with autogene cevumeran with and without atezolizumab in Serial Biopsy Cohort and Biomarker Substudy

Supplementary Table 9. Sampling for high dimensional phenotyping

Supplementary Figure 1. Gating strategy and example plots for multimer gating

Supplementary Table 1. Exposure and disposition of patients treated with autogene cevumeran monotherapy (phase 1a) and with atezolizumab (phase 1b)

Phase 1a exposure and disposition						
	Autogene cevumeran monotherapy dose					
	**25 µg** *n* **= 12**	**38 µg** *n* **= 5**	**50 µg** *n* **= 4**	**75 µg** *n* **= 8**	**100 µg** *n* **= 1**	**All patients** *N* **= 30**
Received autogene cevumeran, *n* (%)	12 (100)	5 (100)	4 (100)	8 (100)	1 (100)	30 (100)
Number of autogene cevumeran doses						
<8	6 (50.0)	2 (40.0)	2 (50.0)	7 (87.5)	0	17 (56.7)
≥8	6 (50.0)	3 (60.0)	2 (50.0)	1 (12.5)	1 (100)	13 (43.3)
Autogene cevumeran dose reduction, *n* (%)	0	1 (20.0)	0	0	1 (100)	2 (6.7)
Autogene cevumeran ongoing, *n* (%)	0	0	0	0	0	0
Autogene cevumeran treatment duration, median (range), days	50 (22–123)	56 (15–128)	40 (15–254)	39.5 (9–69)	56 (56–56)	49.5 (9–254)
Discontinued autogene cevumeran, *n* (%)	12 (100)	5 (100)	4 (100)	8 (100)	1 (100)	30 (100)
Crossover	5 (41.7)	3 (60.0)	3 (75.0)	2 (25.0)	0	13 (43.3)
Disease progression	4 (33.3)	1 (20.0)	1 (25.0)	4 (50.0)	1 (100)	11 (36.7)
Withdrawal by subject	3 (25.0)	1 (20.0)	0	0	0	4 (13.3)
Other	0	0	0	2 (25.0)	0	2 (6.7)
Discontinued autogene cevumeran due to disease progression prior to completing 7 weeks of therapy, *n* (%)	4 (33.3)	1 (20.0)	1 (25.0)	3 (37.5)	0	9 (30.0)

Phase 1b exposure and disposition					
	Autogene cevumeran dose + atezolizumab 1200 mg				
	**15 µg** *n* **= 28**	**25 µg** *n* **= 135**	**38 µg** **n = 11**	**50 µg** *n* **= 9**	**All Patients** *N* **= 183**
Received autogene cevumeran, *n* (%)	28 (100)	131 (97.0)	11 (100)	9 (100)	179 (97.8)
No. of autogene cevumeran doses					
<8	8 (28.6)	54 (41.2)	3 (27.3)	5 (55.6)	70 (39.1)
≥8	20 (71.4)	77 (58.8)	8 (72.7)	4 (44.4)	109 (60.9)
Autogene cevumeran dose reduction, *n* (%)	2 (7.1)	3 (2.2)	1 (9.1)	2 (22.2)	8 (4.4)
Autogene cevumeran ongoing, *n* (%)	2 (7.1)	19 (14.1)	1 (9.1)	0	22 (12.0)
Autogene cevumeran treatment duration, median (range), days	65 (8–778)	57 (1–1072)	64 (35–1092)	36 (1–253)	58 (1–1092)
Discontinued autogene cevumeran, *n* (%)	26 (92.9)	116 (85.9)	10 (90.9)	9 (100)	161 (88.0)
Adverse event	0	7 (5.2)	1 (9.1)	2 (22.2)	10 (5.5)
Death	2 (7.1)	6 (4.4)	0	0	8 (4.4)
Physician decision	1 (3.6)	1 (0.7)	0	1 (11.1)	3 (1.6)
Disease progression	21 (75.0)	99 (73.3)	8 (72.7)	5 (55.6)	133 (72.7)
Withdrawal by subject	1 (3.6)	1 (0.7)	1 (9.1)	0	3 (1.6)
Other	1 (3.6)	2 (1.5)	0	1 (11.1)	4 (2.2)
Discontinued autogene cevumeran due to disease progression prior to completing 7 weeks of therapy, *n* (%)	5 (17.9)	37 (27.4)	2 (18.2)	3 (33.3)	47 (25.7)
Received atezolizumab, *n* (%)	28 (100)	133 (98.5)	11 (100)	9 (100)	181 (98.9)
Number of atezolizumab doses, median (range)	6 (1–40)	4 (1–56)	6 (2–58)	2 (1–15)	4 (1–58)
Atezolizumab ongoing, *n* (%)	2 (7.1)	18 (13.3)	1 (9.1)	0	21 (11.5)
Atezolizumab Treatment Duration, median (range), days	107 (1–820)	65 (1–1178)	106 (21–1239)	22 (1–296)	66 (1–1239)
Discontinued atezolizumab, *n* (%)	26 (92.9)	117 (86.7)	10 (90.9)	9 (100)	162 (88.5)
Adverse event	0	10 (7.4)	1 (9.1)	2 (22.2)	13 (7.1)
Death	2 (7.1)	6 (4.4)	0	0	8 (4.4)
Physician decision	1 (3.6)	1 (0.7)	0	1 (11.1)	3 (1.6)
Disease progression	20 (71.4)	97 (71.9)	8 (72.7)	5 (55.6)	130 (71.0)
Withdrawal by subject	1 (3.6)	2 (1.5)	1 (9.1)	0	4 (2.2)
Other	2 (7.1)	1 (0.7)	0	1 (11.1)	4 (2.2)

Supplementary Table 2. Treatment-related adverse events and individual signs and symptoms of systemic reactions in ≥5% of phase 1a and b patients

Data are reported as *n* (%) of patients who received ≥1 dose of autogene cevumeran or atezolizumab. Events were classified according to the Common Terminology Criteria for Adverse Events, version 5.0.

**Phase 1a treatment-related adverse events**												
	**Autogene cevumeran monotherapy dose**											
	**25 µg *n* = 12**		**38 µg *n* = 5**		**50 µg *n* = 4**		**75 µg *n* = 8**		**100 µg *n* = 1**		**All patients *N* = 30**	
**MedDRA preferred term, *n* (%)**	**Any grade**	**Grade 3–5**	**Any grade**	**Grade 3–5**	**Any grade**	**Grade 3–5**	**Any grade**	**Grade 3–5**	**Any grade**	**Grade 3–5**	**Any grade**	**Grade 3–5**
Infusion-related reaction	8 (66.7)	0	3 (60.0)	0	3 (75.0)	0	3 (37.5)	0	0	0	17 (56.7)	0
Cytokine release syndrome	1 (8.3)	0	1 (20.0)	0	1 (25.0)	0	5 (62.5)	0	1 (100)	1 (100)	9 (30.0)	1 (3.3)
Fatigue	1 (8.3)	0	0	0	1 (25.0)	1 (25.0)	2 (25.0)	1 (12.5)	0	0	4 (13.3)	2 (6.7)
Myalgia	0	0	1 (20.0)	0	0	0	2 (25.0)	0	0	0	3 (10.0)	0
Diarrhea	0	0	0	0	1 (25.0)	0	1 (12.5)	0	0	0	2 (6.7)	0
Nausea	0	0	0	0	0	0	2 (25.0)	0	0	0	2 (6.7)	0
Vomiting	0	0	0	0	0	0	2 (25.0)	0	0	0	2 (6.7)	0

**Phase 1b treatment-related adverse events**										
	**Autogene cevumeran dose + atezolizumab 1200 mg**									
	**15 µg *n* = 28**		**25 µg *n* = 135**		**38 µg *n* = 11**		**50 µg *n* = 9**		**All Patients *N* = 183**	
**MedDRA preferred term, *n* (%)**	**Any grade**	**Grade 3–5**	**Any grade**	**Grade 3–5**	**Any grade**	**Grade 3–5**	**Any grade**	**Grade 3–5**	**Any grade**	**Grade 3–5**
Infusion-related reaction	16 (57.1)	1 (3.6)	80 (59.3)	4 (3.0)	8 (72.7)	1 (9.1)	5 (55.6)	1 (11.1)	109 (59.6)	7 (3.8)
Cytokine release syndrome	1 (3.6)	0	34 (25.2)	0	0	0	3 (33.3)	0	38 (20.8)	0
Fatigue	6 (21.4)	0	27 (20.0)	0	2 (18.2)	0	1 (11.1)	0	36 (19.7)	0
Influenza-like illness	2 (7.1)	0	16 (11.9)	1 (0.7)	4 (36.4)	0	1 (11.1)	0	23 (12.6)	1 (0.5)
Pyrexia	0	0	20 (14.8)	1 (0.7)	1 (9.1)	0	0	0	21 (11.5)	1 (0.5)
Diarrhea	2 (7.1)	0	11 (8.1)	1 (0.7)	2 (18.2)	0	0	0	15 (8.2)	1 (0.5)
Nausea	2 (7.1)	0	11 (8.1)	0	1 (9.1)	0	0	0	14 (7.7)	0
Rash	2 (7.1)	0	10 (7.4)	2 (1.5)	2 (18.2)	0	0	0	14 (7.7)	2 (1.1)
Headache	3 (10.7)	0	9 (6.7)	0	0	0	0	0	12 (6.6)	0
Arthralgia	1 (3.6)	0	10 (7.4)	0	0	0	0	0	11 (6.0)	0
Myalgia	2 (7.1)	0	6 (4.4)	0	1 (9.1)	0	1 (11.1)	0	10 (5.5)	0
Pruritus	2 (7.1)	0	7 (5.2)	0	1 (9.1)	0	0	0	10 (5.5)	0

Supplementary Table 2 (continued). Treatment-related adverse events and individual signs and symptoms of systemic reactions in ≥5% of phase 1a and b patients

**Phase 1a autogene cevumeran monotherapy individual signs and symptoms of systemic reactions**												
	**Autogene cevumeran monotherapy dose**											
	**25 µg *n* = 12**		**38 µg *n* = 5**		**50 µg *n* = 4**		**75 µg *n* = 8**		**100 µg *n* = 1**		**All patients *N* = 30**	
**MedDRA preferred term, *n* (%)**	**Any grade**	**Grade 3–5**	**Any Grade**	**Grade 3–5**	**Any Grade**	**Grade 3–5**	**Any Grade**	**Grade 3–5**	**Any Grade**	**Grade 3–5**	**Any Grade**	**Grade 3–5**
Chills	7 (58.3)	0	4 (80.0)	0	4 (100)	0	8 (100)	0	1 (100)	0	24 (80.0)	0
Pyrexia	5 (41.7)	0	2 (40.0)	0	3 (75.0)	0	5 (62.5)	0	1 (100)	0	16 (53.3)	0
Nausea	3 (25.0)	0	2 (40.0)	0	4 (100)	0	4 (50.0)	0	0	0	13 (43.3)	0
Headache	3 (25.0)	0	1 (20.0)	0	1 (25.0)	0	1 (12.5)	0	0	0	6 (20.0)	0
Vomiting	3 (25.0)	0	1 (20.0)	0	1 (25.0)	0	0	0	0	0	5 (16.7)	0
Hypotension	0	0	1 (20.0)	0	0	0	2 (25.0)	0	1 (100)	1 (100)	4 (13.3)	1 (3.3)
Hypoxia	0	0	1 (20.0)	0	0	0	1 (12.5)	0	1 (100)	1 (100)	3 (10.0)	1 (3.3)
Myalgia	2 (16.7)	0	0	0	0	0	1 (12.5)	0	0	0	3 (10.0)	0
Tachycardia	0	0	0	0	1 (25.0)	0	2 (25.0)	0	0	0	3 (10.0)	0
Neck pain	1 (8.3)	0	1 (20.0)	0	0	0	0	0	0	0	2 (6.7)	0
Sinus tachycardia	1 (8.3)	0	1 (20.0)	0	0	0	0	0	0	0	2 (6.7)	0
Tremor	0	0	1 (20.0)	0	1 (25.0)	0	0	0	0	0	2 (6.7)	0

**Phase 1b autogene cevumeran + atezolizumab 1200 mg individual signs and symptoms of systemic reactions**										
	**Autogene cevumeran dose + atezolizumab 1200 mg**									
	**15 µg *n* = 28**		**25 µg *n* = 135**		**38 µg *n* = 11**		**50 µg *n* = 9**		**All patients *N* = 183**	
**MedDRA preferred term, *n* (%)**	**Any grade**	**Grade 3–5**	**Any grade**	**Grade 3–5**	**Any grade**	**Grade 3–5**	**Any grade**	**Grade 3–5**	**Any grade**	**Grade 3–5**
Pyrexia	10 (35.7)	0	98 (72.6)	5 (3.7)	10 (90.9)	0	6 (66.7)	0	124 (67.8)	5 (2.7)
Chills	12 (42.9)	1 (3.6)	82 (60.7)	2 (1.5)	8 (72.7)	0	7 (77.8)	0	109 (59.6)	4 (2.2)
Nausea	2 (7.1)	0	16 (11.9)	0	2 (18.2)	0	2 (22.2)	0	22 (12.0)	0
Tachycardia	1 (3.6)	0	12 (8.9)	0	2 (18.2)	0	3 (33.3)	0	18 (9.8)	0
Vomiting	1 (3.6)	1 (3.6)	15 (11.1)	0	2 (18.2)	0	0	0	18 (9.8)	1 (0.5)
Headache	3 (10.7)	0	9 (6.7)	0	2 (18.2)	0	0	0	14 (7.7)	0
Hypotension	3 (10.7)	0	7 (5.2)	0	1 (9.1)	0	0	0	11 (6.0)	0
Myalgia	2 (7.1)	0	7 (5.2)	0	1 (9.1)	0	0	0	10 (5.5)	0
Hypertension	1 (3.6)	0	7 (5.2)	0	0	0	2 (22.2)	0	10 (5.5)	0

Supplementary Table 3. Incidence of immune-mediated adverse events observed in phase 1a and phase 1b

Data are reported as *n* (%) of patients who received ≥1 dose of autogene cevumeran or atezolizumab. Immune-mediated adverse events were classified according to the Common Terminology Criteria for Adverse Events, version 5.0.

	**Phase 1a<br>Autogene cevumeran monotherapy<br>*N* = 30**		**Phase 1b<br>Autogene cevumeran + atezolizumab<br>*N* = 183**	
Immune-related AE, *n* (%)	Any grade	Grade 3–5	Any grade	Grade 3–5
Rash	1 (3.3)	0	26 (14.2)	4 (2.2)
Hepatitis (diagnosis and lab)	2 (6.7)	2 (6.7)	22 (12.0)	6 (3.3)
Pancreatitis	0	0	13 (7.1)	6 (3.3)
Hypothyroidism	0	0	10 (5.5)	0
Pneumonitis	0	0	5 (2.7)	2 (1.1)
Colitis	0	0	4 (2.2)	3 (1.6)
Hyperthyroidism	0	0	3 (1.6)	0
Adrenal insufficiency	0	0	1 (0.5)	0
Hypophysitis	0	0	1 (0.5)	0
Nephritis	0	0	1 (0.5)	1 (0.5)
Severe cutaneous reactions	0	0	1 (0.5)	0
Myocarditis	0	0	1 (0.5)	0
Meningo-encephalitis	1 (3.3)	0	0	0
Meningitis	1 (3.3)	0	0	0

Supplementary Table 4. Sampling for immune monitoring by cohort

Autogene cevumeran monotherapy			
**Assay performed**	**Phase 1a**	**Phase 1b**	**Total**
	**Dose Escalation**	**Biomarker substudy**	
Ex vivo IFNγ ELISpot	11	4	15
Post-IVS IFNγ ELISpot	0	5	5
Multiplexed tetramer staining	5	0	5
TCR sequencing analysis	1	5	6

Autogene cevumeran + atezolizumab combination therapy													
**Assay performed**	**Phase 1a**	**Phase 1b**											**Total**
	**Dose escalation**	**Biomarker substudy**	**Dose escalation**		**Dose expansion**								
	**CPI-naive**		**CPI-exp**	**CPI-naive**	**CPI-exp**		**CPI-naïve**					**Serial biopsy**	
			**Various indications**		**Melanoma**	**NSCLC**	**Melanoma**	**NSCLC**	**RCC**	**TNBC**	**UC**		
Ex vivo IFNγ ELISpot	1	15	4	10	4	11	3	3	8	2	7	7	75
Post-IVS IFNγ ELISpot	0	10	0	0	0	0	0	0	0	0	0	0	10
Multiplexed tetramer staining	0	0	0	7	1	4	1	1	1	0	2	0	17
TCR sequencing analysis	0	6	1	0	0	0	0	0	0	0	0	0	7

Supplementary Table 5. List of neoantigen specific TCRs

Patient	TCR ID	TRA VDJ	TRB VDJ	Cell type	HLA Restriction	Target	Type	Frequency post-treatment	Frequency baseline	Frequency Merged Biopsies	Frequency Biopsy 1	Frequency Biopsy 2	Frequency Biopsy 3	Frequency Biopsy 4
13	hTCR-CD8-13-c#2	V27J26C	V24-1J2-1C2	CD8	B*08:01	GALNT6(E579K)	Pre-existing	0.5209	0.0001	n.a.	n.a.	n.a.	n.a.	n.a.
13	hTCR-CD8-13-c#3	V27J26C	V24-1D1J1-1C1	CD8	B*08:01	GALNT6(E579K)	Pre-existing	0.5626	0.0002	n.a.	n.a.	n.a.	n.a.	n.a.
13	hTCR-CD8-13-c#4	V17J9C	V11-2D2J2-7C2	CD8	B*08:01	GALNT6(E579K)	Pre-existing	0.3853	0.0002	n.a.	n.a.	n.a.	n.a.	n.a.
13	hTCR-CD8-13-c#6	V21J39C	V5-5D2J1-5C1	CD8	B*08:01	GALNT6(E579K)	Pre-existing	0.3094	0.0002	n.a.	n.a.	n.a.	n.a.	n.a.
13	hTCR-CD8-13-c#10	V14/DV4J54C	V5-1D1J1-5C1	CD8	B*08:01	GALNT6(E579K)	Pre-existing	0.0836	0.0001	n.a.	n.a.	n.a.	n.a.	n.a.
13	hTCR-CD8-13-c#11	V38-2/DV8J48C	V7-9*03D1J2-7C2	CD8	B*08:01	GALNT6(E579K)	De-novo	0.6307	not detected	n.a.	n.a.	n.a.	n.a.	n.a.
13	hTCR-CD8-13-c#12	V17J28C	V7-6J2-7C2	CD8	B*08:01	GALNT6(E579K)	De-novo	0.6390	not detected	n.a.	n.a.	n.a.	n.a.	n.a.
13	hTCR-CD8-13-c#16	V29/DV5J6C	V5-1D1J2-7C2	CD8	B*08:01	GALNT6(E579K)	De-novo	0.1818	not detected	n.a.	n.a.	n.a.	n.a.	n.a.
13	hTCR-CD8-13-c#17	V19J30C	V7-9*03J2-3C2	CD8	B*08:01	GALNT6(E579K)	De-novo	0.1534	not detected	n.a.	n.a.	n.a.	n.a.	n.a.
13	hTCR-CD8-13-c#21	V14/DV4J49C	V6-1D1J1-5C1	CD8	B*08:01	GALNT6(E579K)	De-novo	0.0323	not detected	n.a.	n.a.	n.a.	n.a.	n.a.
13	hTCR-CD8-13-c#26	V14/DV4J43C	V6-1D1J2-7C2	CD8	B*08:01	GALNT6(E579K)	De-novo	0.0918	not detected	n.a.	n.a.	n.a.	n.a.	n.a.
13	hTCR-CD8-13-c#27	V19J58C	V6-1D2*02J1-5C1	CD8	B*08:01	GALNT6(E579K)	De-novo	0.0895	not detected	n.a.	n.a.	n.a.	n.a.	n.a.
13	hTCR-CD8-13-c#15	V19J8C	V5-6D2J2-3C2	CD8	A*02:05	TTC13(N144Y)	De-novo	0.2280	not detected	n.a.	n.a.	n.a.	n.a.	n.a.
18	hTCR-CD8-18-c#3	V29/DV5J36C	V7-9*03J2-7C2	CD8	A*24:14	C14orf109(R45Q)	Pre-existing	0.1479	0.0001	n.a.	n.a.	n.a.	n.a.	n.a.
18	hTCR-CD8-18-c#21	V3J13*02C	V4-3*02J2-7C2	CD8	A*24:14	C14orf109(R45Q)	De-novo	0.0970	not detected	n.a.	n.a.	n.a.	n.a.	n.a.
18	hTCR-CD8-18-c#2	V22J47C	V19J2-3C2	CD8	A*31:01	CLK3(S270F)	Pre-existing	0.2878	0.0001	n.a.	n.a.	n.a.	n.a.	n.a.
18	hTCR-CD8-18-c#12	V27J52C	V6-1J2-2C2	CD8	A*31:01	CLK3(S270F)	Pre-existing	0.0131	0.0011	n.a.	n.a.	n.a.	n.a.	n.a.
18	hTCR-CD8-18-c#17	V17J42C	V12-4J2-5C2	CD8	B*40:02	DIDO1(T478I)	De-novo	0.1758	not detected	n.a.	n.a.	n.a.	n.a.	n.a.
18	hTCR-CD4-18-c#1	V12-2J18C	V28J1-2C1	CD4	TBD	PLEKHA8(S98R)	Pre-existing	0.0103	0.0001	n.a.	n.a.	n.a.	n.a.	n.a.
18	hTCR-CD8-18-c#22	V25J53C	V10-3J2-1C2	CD8	A*31:01	RNA A	De-novo	0.0827	not detected	n.a.	n.a.	n.a.	n.a.	n.a.
18	hTCR-CD8-18-c#1	V8-1J44C	V7-3J2-5C2	CD8	A*24:14	SVEP1(R136C)	Pre-existing	8.6865	0.0012	n.a.	n.a.	n.a.	n.a.	n.a.
18	hTCR-CD8-18-c#14	V14/DV4*02J48C	V7-9*03J2-7C2	CD8	A*24:14	SVEP1(R136C)	De-novo	0.3863	not detected	n.a.	n.a.	n.a.	n.a.	n.a.
18	hTCR-CD8-18-c#15	V8-3J10C	V4-1J2-2C2	CD8	A*24:14	SVEP1(R136C)	De-novo	0.3597	not detected	n.a.	n.a.	n.a.	n.a.	n.a.
18	hTCR-CD8-18-c#18	V12-2J53C	V6-2J2-7C2	CD8	A*24:14	SVEP1(R136C)	De-novo	0.1679	not detected	n.a.	n.a.	n.a.	n.a.	n.a.
18	hTCR-CD8-18-c#19	V12-1J10C	V2J2-1C2	CD8	A*24:14	SVEP1(R136C)	De-novo	0.1452	not detected	n.a.	n.a.	n.a.	n.a.	n.a.
18	hTCR-CD4-18-c#3	V19J10C	V28J2-1C2	CD4	TBD	SVEP1(R136C)	Pre-existing	0.0051	0.0001	n.a.	n.a.	n.a.	n.a.	n.a.

18	hTCR-CD4-18-c#14	V26-1J41C	V24-1J2-1C2	CD4	TBD	SVEP1(R136C)	De-novo	0.0357	not detected	n.a.	n.a.	n.a.	n.a.	n.a.
18	hTCR-CD4-18-c#18	V19J17C	V28J2-3C2	CD4	TBD	SVEP1(R136C)	De-novo	0.0196	not detected	n.a.	n.a.	n.a.	n.a.	n.a.
18	hTCR-CD8-18-c#6a1	V8-3J6C	V7-9*03J2-7C2	CD8	A*24:14	TP53I3(D142N)	Pre-existing	0.0646	0.0012	n.a.	n.a.	n.a.	n.a.	n.a.
31	hTCR-CD8-31-c#1	V13-1J43C	V10-3J2-1C2	CD8	B*37:01	DCHS1(R215Q)	Pre-existing	5.6205	0.0019	not detected	n.a.	n.a.	n.a.	n.a.
31	hTCR-CD4-31-c#22a2	V24J48C	V6-5J2-1C2	CD4	DRA1*01:01 + DRB1*11:01	ESF1(R12W)	De-novo	0.0009	not detected	not detected	n.a.	n.a.	n.a.	n.a.
31	hTCR-CD8-31-c#3	V13-2J30C	V6-5J1-1C1	CD8	TBD	KIAA0753(Q794K)	De-novo	0.4613	not detected	not detected	n.a.	n.a.	n.a.	n.a.
31	hTCR-CD8-31-c#4	V12-3J40C	V7-9*03J2-7C2	CD8	TBD	KIAA0753(Q794K)	De-novo	0.3888	not detected	not detected	n.a.	n.a.	n.a.	n.a.
31	hTCR-CD8-31-c#9a1	V38-1J23C	V4-3*02J1-2C1	CD8	B*58:01	KIAA0753(Q794K)	De-novo	0.1027	not detected	not detected	n.a.	n.a.	n.a.	n.a.
31	hTCR-CD8-31-c#9a2	V26-1J27C	V4-3*02J1-2C1	CD8	B*58:01	KIAA0753(Q794K)	De-novo	0.1027	not detected	not detected	n.a.	n.a.	n.a.	n.a.
31	hTCR-CD8-31-c#8	V39J26C	V7-9J1-1C1	CD8	B*58:01	NARG2(G11E)	De-novo	0.1193	not detected	not detected	n.a.	n.a.	n.a.	n.a.
31	hTCR-CD4-31-c#7	V5J31C	V4-2J2-1C2	CD4	DRA1*01:01 + DRB1*11:03	RNA B	Pre-existing	0.0018	0.0014	not detected	n.a.	n.a.	n.a.	n.a.
31	hTCR-CD4-31-c#10.1	V9-2*02J34C	V7-7J1-1C1	CD4	DQA1*05:05+DQB1*03:01	RNA B	De-novo	0.0350	not detected	not detected	n.a.	n.a.	n.a.	n.a.
31	hTCR-CD4-31-c#11.1	V36/DV7J45C	V20-1*02J2-7C2	CD4	DQA1*05:05+DQB1*03:01	RNA B	De-novo	0.0267	not detected	not detected	n.a.	n.a.	n.a.	n.a.
31	hTCR-CD4-31-c#14	V20J54C	V20-1*02J2-1C2	CD4	DQA1*05:05+DQB1*03:01	RNA B	De-novo	0.0223	not detected	not detected	n.a.	n.a.	n.a.	n.a.
31	hTCR-CD4-31-c#19a1	V12-2J49C	V15*02J1-1C1	CD4	DRA1*01:01 + DRB1*11:03	RNA B	De-novo	0.0066	not detected	not detected	n.a.	n.a.	n.a.	n.a.
31	hTCR-CD8-31-c#2	V12-3J24*02C	V28J1-1C1	CD8	C*06:02	SHROOM2(R751Q)	De-novo	1.3391	not detected	not detected	n.a.	n.a.	n.a.	n.a.
31	hTCR-CD8-31-c#5	V23/DV6J20C	V19J2-3C2	CD8	C*06:02	SHROOM2(R751Q)	De-novo	0.2856	not detected	not detected	n.a.	n.a.	n.a.	n.a.
31	hTCR-CD8-31-c#6	V38-2/DV8J57C	V13*02J1-1C1	CD8	C*06:02	SHROOM2(R751Q)	De-novo	0.2752	not detected	not detected	n.a.	n.a.	n.a.	n.a.
31	hTCR-CD4-31-c#19a2	V22J36C	V15*02J1-1C1	CD4	DRA1*01:01 + DRB1*11:03	SHROOM2(R751Q)	De-novo	0.0066	not detected	not detected	n.a.	n.a.	n.a.	n.a.
31	hTCR-CD4-31-c#21	V38-1J52C	V12-3J1-1C1	CD4	DRA1*01:01 + DRB1*11:03	SHROOM2(R751Q)	De-novo	0.0033	not detected	not detected	n.a.	n.a.	n.a.	n.a.
34	hTCR-CD8-34-c#1	V12-3J6C	V12-3J2-2C2	CD8	B*08:01	LIPC(M103K)	Pre-existing	9.8832	0.0002	n.a.	n.a.	n.a.	n.a.	n.a.
34	hTCR-CD8-34-c#10	V10J16C	V20-1*02J2-7C2	CD8	B*08:01	LIPC(M103K)	Pre-existing	0.0095	0.0004	n.a.	n.a.	n.a.	n.a.	n.a.
34	hTCR-CD8-34-c#11	V12-3J6C	V12-3J2-1C2	CD8	B*08:01	LIPC(M103K)	De-novo	2.8447	not detected	n.a.	n.a.	n.a.	n.a.	n.a.
34	hTCR-CD8-34-c#12	V12-3J6C	V12-3J2-2C2	CD8	B*08:01	LIPC(M103K)	De-novo	2.3478	not detected	n.a.	n.a.	n.a.	n.a.	n.a.
34	hTCR-CD8-34-c#16	V12-3J53C	V12-3J1-2C1	CD8	B*08:01	LIPC(M103K)	De-novo	1.2810	not detected	n.a.	n.a.	n.a.	n.a.	n.a.
34	hTCR-CD8-34-c#17	V12-3J6C	V12-3J1-2C1	CD8	B*08:01	LIPC(M103K)	De-novo	1.2457	not detected	n.a.	n.a.	n.a.	n.a.	n.a.
34	hTCR-CD8-34-c#18	V19J50C	V12-4J2-3C2	CD8	A*11:01	LIPC(M103K)	De-novo	0.7760	not detected	n.a.	n.a.	n.a.	n.a.	n.a.
34	hTCR-CD8-34-c#19	V19J37C	V10-3J1-1C1	CD8	A*11:01	LIPC(M103K)	De-novo	0.7728	not detected	n.a.	n.a.	n.a.	n.a.	n.a.
34	hTCR-CD8-34-c#20	V19J42C	V27J2-6C2	CD8	A*11:01	LIPC(M103K)	De-novo	0.7727	not detected	n.a.	n.a.	n.a.	n.a.	n.a.
34	hTCR-CD8-34-c#15	V26-1J49C	V20-1*02J2-1C2	CD8	TBD	MON1A(V627I)	De-novo	1.3129	not detected	n.a.	n.a.	n.a.	n.a.	n.a.

34	hTCR-CD8-34-c#13	V19J28C	V5-1J2-1C2	CD8	B*08:01	ZBTB5(V41M)	De-novo	1.9854	not detected	n.a.	n.a.	n.a.	n.a.	n.a.
37	hTCR-CD8-37-c#7	V8-4J9C	V3-1J1-6C1	CD8	TBD	RNA B	De-novo	0.0492	not detected	0.0675	0.0520	0.0898	0.0643	0.0590
37	hTCR-CD8-37-c#2.2	V8-2J40C	V29-1D1J2-7C2	CD8	TBD	TMEM232(Y550S)	De-novo	0.1126	not detected	0.2291	0.0786	0.5620	0.0801	0.1066
37	hTCR-CD8-37-c#8	V8-2J48C	V7-2J2-7C2	CD8	TBD	TMEM232(Y550S)	De-novo	0.0462	not detected	0.0604	0.0595	not detected	0.1040	0.0922
38	hTCR-CD8-38-c#2	V19J44C	V13J2-1C2	CD8	A*02:01	KIAA0100(T1959A)	De-novo	1.2018	not detected	n.a.	n.a.	n.a.	n.a.	n.a.
38	hTCR-CD8-38-c#5.1	V22J10C	V27J2-7C2	CD8	A*02:01	KIAA0100(T1959A)	De-novo	1.2921	not detected	2.4646	n.a.	n.a.	n.a.	n.a.
38	hTCR-CD8-38-c#1	V8-2J4C	V19J2-6C2	CD8	TBD	RNA A	De-novo	0.9283	not detected	0.0122	n.a.	n.a.	n.a.	n.a.
38	hTCR-CD8-38-c#3.1	V21J41C	V7-9*03J2-7C2	CD8	TBD	RNA A	De-novo	1.4027	not detected	0.0251	n.a.	n.a.	n.a.	n.a.
38	hTCR-CD8-38-c#4	V19J39C	V7-9J2-3C2	CD8	TBD	RNA A	De-novo	0.7011	not detected	0.0319	n.a.	n.a.	n.a.	n.a.
38	hTCR-CD8-38-c#10	V19J20C	V20-1J2-3C2	CD8	TBD	RNA A	De-novo	0.6446	not detected	n.a.	n.a.	n.a.	n.a.	n.a.
38	hTCR-CD8-38-c#12	V19J20C	V20-1J2-3C2	CD8	TBD	RNA A	De-novo	0.2517	not detected	n.a.	n.a.	n.a.	n.a.	n.a.
38	hTCR-CD8-38-c#14	V17J29C	V7-9*03J2-1C2	CD8	TBD	RNA A	De-novo	0.2091	not detected	0.0206	n.a.	n.a.	n.a.	n.a.
38	hTCR-CD8-38-c#18.1	V17J29C	V7-9*03J2-7C2	CD8	B*07:02	RNA A	Pre-existing	0.1802	0.0007	0.0002	n.a.	n.a.	n.a.	n.a.
38	hTCR-CD8-38-c#18.2	V17J29C	V7-9*03J2-7C2	CD8	B*07:02	RNA A	Pre-existing	0.1802	0.0007	0.0002	n.a.	n.a.	n.a.	n.a.
38	hTCR-CD8-38-c#18.3	V17J29C	V7-9*03J2-7C2	CD8	B*07:02	RNA A	Pre-existing	0.1802	0.0007	0.0002	n.a.	n.a.	n.a.	n.a.
38	hTCR-CD8-38-c#22	V17J29C	V7-9*03J2-1C2	CD8	B*07:02	RNA A	De-novo	0.1746	not detected	n.a.	n.a.	n.a.	n.a.	n.a.
40	hTCR-CD8-40-c#6	V16J28C	V9J2-5C2	CD8	B*18:01	MTRR(C340F)	Pre-existing	0.1052	0.0010	0.1422	0.1376	0.1473	n.a.	n.a.
40	hTCR-CD8-40-c#8	V39J30C	V9J1-1C1	CD8	A*01:01	RNA A	Pre-existing	0.0043	0.0001	0.0002	not detected	0.0003	n.a.	n.a.
40	hTCR-CD8-40-c#2.2	V38-2/DV8J30C	V7-9J2-7C2	CD8	B*18:01	RNA B	Pre-existing	1.1032	0.0024	0.1089	0.1023	0.1176	n.a.	n.a.
40	hTCR-CD8-40-c#3	V38-1J12C	V7-9J2-7C2	CD8	B*18:01	RNA B	Pre-existing	0.0576	0.0001	0.0156	0.0190	0.0121	n.a.	n.a.
40	hTCR-CD8-40-c#10	V1-2J31C	V6-2J1-1C1	CD8	B*18:01	RNA B	Pre-existing	0.0337	0.0018	0.0056	not detected	0.0118	n.a.	n.a.
40	hTCR-CD8-40-c#11.1	V38-2/DV8J30C	V7-9J2-7C2	CD8	B*18:01	RNA B	De-novo	1.8065	not detected	0.0865	0.0715	0.1020	n.a.	n.a.
40	hTCR-CD8-40-c#1	V38-1J12C	V7-9J2-7C2	CD8	B*18:01	TMEM2(R295M)	Pre-existing	0.4650	0.0003	0.0300	0.0365	0.0232	n.a.	n.a.
40	hTCR-CD8-40-c#2.1	V38-2/DV8J30C	V7-9J2-7C2	CD8	B*18:01	TMEM2(R295M)	Pre-existing	1.1032	0.0024	0.1089	0.1023	0.1176	n.a.	n.a.
40	hTCR-CD8-40-c#5	V39J42C	V9J2-2C2	CD8	B*18:01	TMEM2(R295M)	Pre-existing	0.2302	0.0016	0.0405	0.0590	0.0213	n.a.	n.a.
40	hTCR-CD8-40-c#7	V8-6J53C	V4-1J1-4C1	CD8	B*18:01	TMEM2(R295M)	Pre-existing	0.1007	0.0019	0.1032	0.1282	0.0773	n.a.	n.a.
40	hTCR-CD8-40-c#9	V29/DV5J33C	V4-1J2-7C2	CD8	B*18:01	TMEM2(R295M)	Pre-existing	0.0159	0.0005	0.0015	not detected	0.0031	n.a.	n.a.
40	hTCR-CD8-40-c#12	V38-1J44C	V10-3J2-7C2	CD8	B*18:01	TMEM2(R295M)	De-novo	1.7325	not detected	0.0158	not detected	0.0321	n.a.	n.a.
40	hTCR-CD8-40-c#13	V12-2J3C	V7-3D1J1-1C1	CD8	B*18:01	TMEM2(R295M)	De-novo	1.4969	not detected	0.1680	0.2046	0.1302	n.a.	n.a.
40	hTCR-CD8-40-c#14	V24J11C	V9D1J2-7C2	CD8	B*18:01	TMEM2(R295M)	De-novo	0.9320	not detected	0.1179	0.1393	0.0961	n.a.	n.a.
40	hTCR-CD8-40-c#15	V8-1J8C	V7-8J2-1C2	CD8	B*18:01	TMEM2(R295M)	De-novo	0.6871	not detected	0.0658	0.0952	0.0351	n.a.	n.a.
40	hTCR-CD8-40-c#16	V38-2/DV8J30C	V7-9J2-7C2	CD8	B*18:01	TMEM2(R295M)	De-novo	0.5784	not detected	0.0864	0.1166	0.0560	n.a.	n.a.

40	hTCR-CD8-40-c#17	V34J40C	V4-1J2-7C2	CD8	B*18:01	TMEM2(R295M)	De-novo	0.4797	not detected	0.0715	0.0863	0.0563	n.a.	n.a.
40	hTCR-CD8-40-c#19	V12-1J39C	V5-1J2-7C2	CD8	B*18:01	TMEM2(R295M)	De-novo	0.3514	not detected	0.0216	0.0118	0.0318	n.a.	n.a.
40	hTCR-CD8-40-c#20	V29/DV5J20C	V4-1D1J1-2C1	CD8	B*18:01	TMEM2(R295M)	De-novo	0.2969	not detected	0.0078	not detected	0.0161	n.a.	n.a.
42	hTCR-CD8-42-c#13a1	V4J22C	V11-2J2-5C2	CD8	A*02:01	USP34(S3003P)	De-novo	1.9935	not detected	not detected	n.a.	n.a.	n.a.	n.a.
42	hTCR-CD8-42-c#13a2	V4J3C	V11-2J2-5C2	CD8	A*02:01	USP34(S3003P)	De-novo	1.9935	not detected	not detected	n.a.	n.a.	n.a.	n.a.
42	hTCR-CD8-42-c#15	V35J57C	V20-1J2-1C2	CD8	A*02:01	USP34(S3003P)	De-novo	0.6524	not detected	not detected	n.a.	n.a.	n.a.	n.a.
42	hTCR-CD8-42-c#18	V8-4J16C	V4-1J2-1C2	CD8	A*02:01	USP34(S3003P)	De-novo	0.5927	not detected	not detected	n.a.	n.a.	n.a.	n.a.
42	hTCR-CD8-42-c#20	V5J23C	V5-1J2-7C2	CD8	A*02:01	USP34(S3003P)	De-novo	0.3599	not detected	not detected	n.a.	n.a.	n.a.	n.a.
42	hTCR-CD8-42-c#21	V12-2J12C	V4-3*02J2-1C2	CD8	A*02:01	USP34(S3003P)	De-novo	0.3407	not detected	not detected	n.a.	n.a.	n.a.	n.a.
43	hTCR-CD8-43-c#1	V38-2/DV8J56C	V7-9J2-7C2	CD8	B*40:01	RNA B	Pre-existing	0.2014	0.0011	not detected	n.a.	n.a.	n.a.	n.a.
43	hTCR-CD8-43-c#2	V12-3J26C	V5-1J1-5C1	CD8	A*02:01	RNA B	Pre-existing	0.0417	0.0005	not detected	n.a.	n.a.	n.a.	n.a.
43	hTCR-CD8-43-c#6	V17J41C	V7-8J1-1C1	CD8	A*02:01	RNA B	De-novo	0.7922	not detected	0.0960	n.a.	n.a.	n.a.	n.a.
43	hTCR-CD8-43-c#7	V21J13C	V7-7J2-2C2	CD8	B*40:01	RNA B	De-novo	0.7735	not detected	not detected	n.a.	n.a.	n.a.	n.a.
43	hTCR-CD8-43-c#8	V3J17C	V9D1J1-2C1	CD8	A*02:01	RNA B	De-novo	0.6196	not detected	not detected	n.a.	n.a.	n.a.	n.a.
43	hTCR-CD8-43-c#9	V3J20C	V6-5J1-1C1	CD8	B*40:01	RNA B	De-novo	0.5878	not detected	not detected	n.a.	n.a.	n.a.	n.a.
43	hTCR-CD8-43-c#10	V8-3J16C	V28J2-7C2	CD8	B*40:01	RNA B	De-novo	0.5362	not detected	not detected	n.a.	n.a.	n.a.	n.a.
43	hTCR-CD8-43-c#11	V3J39C	V9J2-2C2	CD8	A*02:01	RNA B	De-novo	0.3828	not detected	not detected	n.a.	n.a.	n.a.	n.a.
43	hTCR-CD8-43-c#12	V3J39C	V9J2-2C2	CD8	A*02:01	RNA B	De-novo	0.2143	not detected	0.0524	n.a.	n.a.	n.a.	n.a.
43	hTCR-CD8-43-c#13	V38-2/DV8J39C	V7-9J1-1C1	CD8	B*40:01	RNA B	De-novo	0.1483	not detected	not detected	n.a.	n.a.	n.a.	n.a.
43	hTCR-CD8-43-c#14	V12-3J17C	V7-2J1-5C1	CD8	A*02:01	RNA B	De-novo	0.1383	not detected	not detected	n.a.	n.a.	n.a.	n.a.
43	hTCR-CD8-43-c#15	V41J32C	V9J2-2C2	CD8	A*02:01	RNA B	De-novo	0.1214	not detected	not detected	n.a.	n.a.	n.a.	n.a.
44	hTCR-CD4-44-c#15	V26-1J42C	V5-6J2-3C2	CD4	DQA03:01 + DQB03:02	IQCJ-SCHIP1(D75H)	De-novo	0.0678	not detected	0.0017	n.a.	n.a.	n.a.	n.a.
44	hTCR-CD4-44-c#13	V35J33C	V5-1J2-4C2	CD4	DRA01:01 + DRB03:01	MAP3K4(G1362R)	De-novo	0.0846	not detected	0.0147	n.a.	n.a.	n.a.	n.a.
44	hTCR-CD8-44-c#12a1	V35J31C	V5-1J2-1C2	CD8	B*40:01	OR5C1(G45E)	De-novo	4.1082	not detected	0.0582	n.a.	n.a.	n.a.	n.a.
44	hTCR-CD8-44-c#12a2	V13-1J13*02C	V5-1J2-1C2	CD8	B*40:01	OR5C1(G45E)	De-novo	4.1082	not detected	0.0582	n.a.	n.a.	n.a.	n.a.
44	hTCR-CD8-44-c#20	V13-2J39C	V2J2-7C2	CD8	C*07:01	PASK(R225C)	De-novo	1.3366	not detected	0.1480	n.a.	n.a.	n.a.	n.a.
44	hTCR-CD8-44-c#2	V19J54C	V9J1-2C1	CD8	B*40:01	SLC23A2(P97S)	Pre-existing	0.2120	0.0001	0.2391	n.a.	n.a.	n.a.	n.a.
44	hTCR-CD8-44-c#3	V1-2J4C	V6-5J1-1C1	CD8	B*40:01	SLC23A2(P97S)	Pre-existing	0.5295	0.0005	0.0640	n.a.	n.a.	n.a.	n.a.
44	hTCR-CD8-44-c#5	V12-2J34C	V6-1J2-1C2	CD8	B*40:01	SLC23A2(P97S)	Pre-existing	0.1167	0.0003	0.0107	n.a.	n.a.	n.a.	n.a.
44	hTCR-CD8-44-c#6	V29/DV5J34C	V14J2-3C2	CD8	B*40:01	SLC23A2(P97S)	Pre-existing	0.0870	0.0003	0.0634	n.a.	n.a.	n.a.	n.a.
44	hTCR-CD8-44-c#7	V8-4J40C	V12-4J1-2C1	CD8	B*40:01	SLC23A2(P97S)	Pre-existing	0.2562	0.0009	0.1282	n.a.	n.a.	n.a.	n.a.
44	hTCR-CD8-44-c#10	V8-6*02J40C	V5-5J2-3C2	CD8	B*40:01	SLC23A2(P97S)	Pre-existing	0.1024	0.0008	0.1288	n.a.	n.a.	n.a.	n.a.

44	hTCR-CD8-44-c#18	V17J11C	V7-9*03J2-3C2	CD8	B*40:01	TBC1D22A(R100H)	De-novo	1.8599	not detected	0.0780	n.a.	n.a.	n.a.	n.a.
76	hTCR-CD8-76-c#1	V14/DV4*02J52C	V30J1-3C1	CD8	A*02:01	RNA A	Pre-existing	0.3290	0.0001	0.0017	0.0033	0.0006	0.0014	n.a.
76	hTCR-CD8-76-c#2a1	V20J52C	V6-5J1-1C1	CD8	A*02:01	RNA A	Pre-existing	0.1702	0.0002	0.0020	0.0032	not detected	0.0032	n.a.
76	hTCR-CD8-76-c#3	V10J29C	V6-5J2-7C2	CD8	A*02:01	RNA A	Pre-existing	0.0710	0.0001	0.0013	0.0002	0.0033	not detected	n.a.
76	hTCR-CD8-76-c#4	V35J43C	V30J1-5C1	CD8	A*02:01	RNA A	Pre-existing	0.0497	0.0001	0.0003	0.0010	not detected	not detected	n.a.
76	hTCR-CD8-76-c#5	V10J29C	V6-5J2-7C2	CD8	A*02:01	RNA A	Pre-existing	0.1642	0.0006	0.0046	0.0032	0.0078	0.0020	n.a.
76	hTCR-CD8-76-c#6.1	V5J23C	V30J1-3C1	CD8	A*02:01	RNA A	De-novo	0.5129	not detected	0.0065	0.0073	0.0081	0.0036	n.a.
76	hTCR-CD8-76-c#6.2	V19J54C	V30J1-3C1	CD8	A*02:01	RNA A	De-novo	0.5129	not detected	0.0065	0.0073	0.0081	0.0036	n.a.
76	hTCR-CD8-76-c#6.3	V5J23C	V30J1-3C1	CD8	A*02:01	RNA A	De-novo	0.5129	not detected	0.0065	0.0073	0.0081	0.0036	n.a.
76	hTCR-CD8-76-c#8	V5J31C	V30J2-1C2	CD8	A*02:01	RNA A	De-novo	0.3887	not detected	0.0017	0.0016	0.0029	0.0004	n.a.
76	hTCR-CD8-76-c#9	V10J23C	V30J2-7C2	CD8	A*02:01	RNA A	De-novo	0.2834	not detected	0.0028	0.0032	0.0044	0.0003	n.a.
76	hTCR-CD8-76-c#10	V5J23C	V30J1-3C1	CD8	A*02:01	RNA A	De-novo	0.2220	not detected	0.0006	0.0015	not detected	0.0004	n.a.
76	hTCR-CD8-76-c#11	V17J9C	V25-1J2-2C2	CD8	A*02:01	RNA A	De-novo	0.2132	not detected	0.0035	0.0046	0.0028	0.0033	n.a.
76	hTCR-CD8-76-c#14	V13-1J49C	V30J2-5C2	CD8	A*02:01	RNA A	De-novo	0.1593	not detected	0.0013	0.0014	not detected	0.0030	n.a.
76	hTCR-CD8-76-c#15.2	V5J23C	V30J2-1C2	CD8	A*02:01	RNA A	De-novo	0.1555	not detected	0.0012	0.0024	not detected	0.0014	n.a.
85	hTCR-CD4-85-c#10	V9-2J17C	V6-5D1J2-2C2	CD4	DQA1*05:01 + DQB1:02:01	KDM5C(I494F)	De-novo	0.0002	not detected	n.a.	n.a.	n.a.	n.a.	n.a.
85	hTCR-CD8-85-c#10	V19J40C	V5-6J2-2C2	CD8	A*0101	KDM5C(I494F)	De-novo	0.3444	not detected	0.1166	0.0724	0.1703	0.1021	n.a.
85	hTCR-CD8-85-c#6	V1-1J31C	V18J2-5C2	CD8	B*08:01	NDUFV1(F442L)	De-novo	0.9552	not detected	0.3671	0.3558	0.5044	0.2527	n.a.
85	hTCR-CD8-85-c#15	V29/DV5J45C	V25-1J2-5C2	CD8	C*07:01	RNA A	De-novo	0.1580	not detected	0.0242	0.0204	0.0305	0.0210	n.a.
85	hTCR-CD8-85-c#7	V39J49C	V9J1-1C1	CD8	B*08:01	TFR2(M705R)	De-novo	0.5159	not detected	0.1005	0.0626	0.1708	0.0659	n.a.
85	hTCR-CD8-85-c#12	V19J13C	V7-6J2-1C2	CD8	B*08:01	UTP20(L699F)	De-novo	0.2387	not detected	0.0697	0.0728	0.1319	0.0098	n.a.
85	hTCR-CD8-85-c#13	V5J39C	V18J1-1C1	CD8	B*08:01	UTP20(L699F)	De-novo	0.2134	not detected	0.0651	0.0576	0.0571	0.0785	n.a.
85	hTCR-CD8-85-c#8	V8-3J26C	V7-9J1-5C1	CD8	B*08:01	VPS13B(T2323P)	De-novo	0.4425	not detected	0.1803	0.0109	0.3374	0.1669	n.a.
90	hTCR-CD8-90-c#3	V21J29C	V5-5J1-2C1	CD8	B57:01	APC	Pre-existing	0.0787	0.0016	0.0947	0.1623	0.0661	0.0562	n.a.
90	hTCR-CD8-90-c#5	V12-1J16C	V3-1J1-1C1	CD8	B57:01	APC	De-novo	2.1937	not detected	1.1435	1.3412	1.0735	1.0195	n.a.
90	hTCR-CD8-90-c#7	V12-2J18C	V12-4J2-1C2	CD8	A32:01	KIAA1211(R582W)	De-novo	1.7566	not detected	0.7654	1.0527	0.4705	0.7278	n.a.
90	hTCR-CD8-90-c#10	V8-2J9C	V27J2-7C2	CD8	A32:01	KIAA1211(R582W)	De-novo	0.7369	not detected	0.2338	0.1914	0.1917	0.3036	n.a.
90	hTCR-CD8-90-c#13	V8-2J9C	V27D1J1-5C1	CD8	A32:01	KIAA1211(R582W)	De-novo	0.6281	not detected	2.1222	2.2459	1.5835	2.4044	n.a.
90	hTCR-CD8-90-c#2	V4J39C	V2J2-7C2	CD8	B57:01	MIPOL1(E137G)	Pre-existing	0.0935	0.0013	0.0369	0.0351	0.0363	0.0393	n.a.
90	hTCR-CD8-90-c#4	V38-1J49C	V2J2-3C2	CD8	B57:01	MIPOL1(E137G)	De-novo	3.4447	not detected	0.9155	1.0060	0.6100	1.0611	n.a.
90	hTCR-CD8-90-c#6	V39J43C	V6-6J1-1C1	CD8	B57:01	MIPOL1(E137G)	De-novo	1.8379	not detected	0.4148	0.4448	0.3237	0.4557	n.a.
90	hTCR-CD8-90-c#8	V5J23C	V9J2-3C2	CD8	B57:01	MIPOL1(E137G)	De-novo	1.5451	not detected	0.3311	0.3267	0.3706	0.3080	n.a.

90	hTCR-CD8-90-c#9	V21J54C	V6-5J2-4C2	CD8	B57:01	MIPOL1(E137G)	De-novo	1.4616	not detected	0.4101	0.4921	0.3032	0.4178	n.a.
90	hTCR-CD8-90-c#11	V14/DV4J52C	V3-1J2-7C2	CD8	B57:01	MIPOL1(E137G)	De-novo	0.6670	not detected	0.5602	0.6918	0.1726	0.7266	n.a.
90	hTCR-CD8-90-c#12	V20J11C	V12-3J2-1C2	CD8	B57:01	MIPOL1(E137G)	De-novo	0.6285	not detected	0.1531	0.2048	0.0790	0.1628	n.a.

n.a. Sample not available

Supplementary Table 6. Clinical activity in patients treated with autogene cevumeran monotherapy and with atezolizumab

	N	ORR, *n* (%)	Median PFS, months	Median OS, months	Median DOR, months
Phase 1a dose escalation	30	1 (3.3)	1.9	11.9	21.4
Phase 1b dose escalation	30	2 (6.7)	2.7	17.1	9.1
Phase 1b NSCLC CPI-experienced	28	1 (3.6)	2.6	11.7	2.8
Phase 1b melanoma CPI-experienced	8	0	4.0	20	15.5
Phase 1b melanoma CPI-naïve	9	3 (33.3)	1.4	NE	NE
Phase 1b NSCLC CPI naïve	10	1 (10.0)	2.4	23.2	17.5
Phase 1b UC CPI-naïve	11	2 (18.2)	2.8	12.6	9.2
Phase 1b RCC CPI-naïve	12	4 (33.3)	8.5	NE	18.0
Phase 1b TNBC CPI-naïve	21	0	1.2	6.2	1.6
Phase Ib serial biopsy	19	2 (10.5)	1.4	11.6	NE
Biomarker substudy	35	0	1.4	9	NE

CPI, checkpoint inhibitor; DOR, duration of response: NE, non-evaluable; NSCLC, non–small cell lung cancer; ORR, objective response rate; OS, overall survival; PFS, progression-free survival; RCC, renal cell carcinoma; TNBC, triple-negative breast cancer; UC, urothelial cancer.

Supplementary Table 7. Clinical activity in patients treated with autogene cevumeran with atezolizumab in phase 1b dose expansion

CPI experience	Autogene cevumeran dose + atezolizumab 1200 mg	Cohort	Prior therapies, median (range)	PD-L1 expression, *n* (%)			Best overall response, *n* (%)				
				<5%	≥5%	Missing	ORR	CR	PR	SD	PD
CPI-experienced	15 µg	NSCLC *n* = 20	3 (1–7)	8 (40.0)	8 (40.0)	4 (20.0)	0	0	0	13 (65.0)	6 (30.0)
	25 µg	NSCLC *n* = 8	4 (3–8)	4 (50.0)	4 (50.0)	0	1(12.5)	0	1 (12.5)	2 (25.0)	4 (50.0)
	15 µg	Melanoma *n* = 8	2.5 (1–10)	5 (62.5)	1 (12.5)	2 (25.0)	0	0	0	6 (75.0)	1 (12.5)
CPI-naïve	25 µg	NSCLC *n* = 10	1.5 (1–5)	8 (80.0)	0	2 (20.0)	1 (10.0)	0	1 (10.0)	4 (40.0)	4 (40.0)
		UC *n* = 11	1 (1–3)	8 (72.7)	3 (27.3)	0	2 (18.2)	1 (9.1)	1 (9.1)	2 (18.2)	4 (36.4)
		TNBC *n* = 21	3 (1–10)	15 (71.4)	4 (19.0)	2 (9.5)	0	0	0	6 (28.6)	11 (52.4)
		RCC *n* = 12	1 (1–1)	9 (75.0)	2 (16.7)	1 (8.3)	4 (33.3)	0	4 (33.3)	6 (50.0)	1 (8.3)
		Melanoma *n* = 9	1 (1–2)	9 (100)	0	0	3 (33.3)	1 (11.1)	2 (22.2)	1 (11.1)	5 (55.6)

CPI, checkpoint inhibitor; CR, complete response; NSCLC, non–small cell lung cancer; ORR, overall response rate; PD-L1, programmed death ligand 1; PD, progressive disease; PR, partial response; RCC, renal cell carcinoma; SD, stable disease; TNBC, triple-negative breast cancer; UC, urothelial cancer.

Supplementary Table 8. Clinical activity in patients treated with autogene cevumeran with and without atezolizumab in Serial Biopsy Cohort and Biomarker Substudy

Autogene cevumeran dose + atezolizumab 1200 mg	Cohort	CPI-experienced, *n* (%)	Prior therapies, median (range)	PD-L1 expression, *n* (%)			Best overall response, *n* (%)				
				<5%	≥5%	Missing	ORR	CR	PR	SD	PD
25 µg	Serial biopsy *n* = 19	5 (26.3)	4 (1–9)	11 (57.9)	3 (15.8)	5 (26.3)	2 (10.5)	1 (5.3)	1 (5.3)	6 (31.6)	10 (52.6)
25 µg	Biomarker substudy *n* = 35	22 (62.8)	4 (1–7)	12 (34.2)	6 (17.1)	17 (48.6)	0	0	0	12 (34.3)	18 (51.4)

CPI, checkpoint inhibitor; CR, complete response; ORR, overall response rate; PD-L1, programmed death ligand1; PD, progressive disease; PR, partial response; SD, stable disease.

Supplementary Table 9. Sampling for high dimensional phenotyping

	Induction Cycles				Maintenance Cycles			
Patient ID	Baseline	D22	D43	D64	D106	D127	D148	D232
55			Y		Y		Y	
18		Y			Y			Y
7		Y		Y	Y			
13		Y			Y			Y
21		Y			Y		Y	
8	Y		Y	Y				
57			Y		Y		Y	
11		Y			Y		Y	
94		Y		Y	Y			
54		Y				Y		

Supplementary Figure 1. Gating strategy and example plots for multimer gating

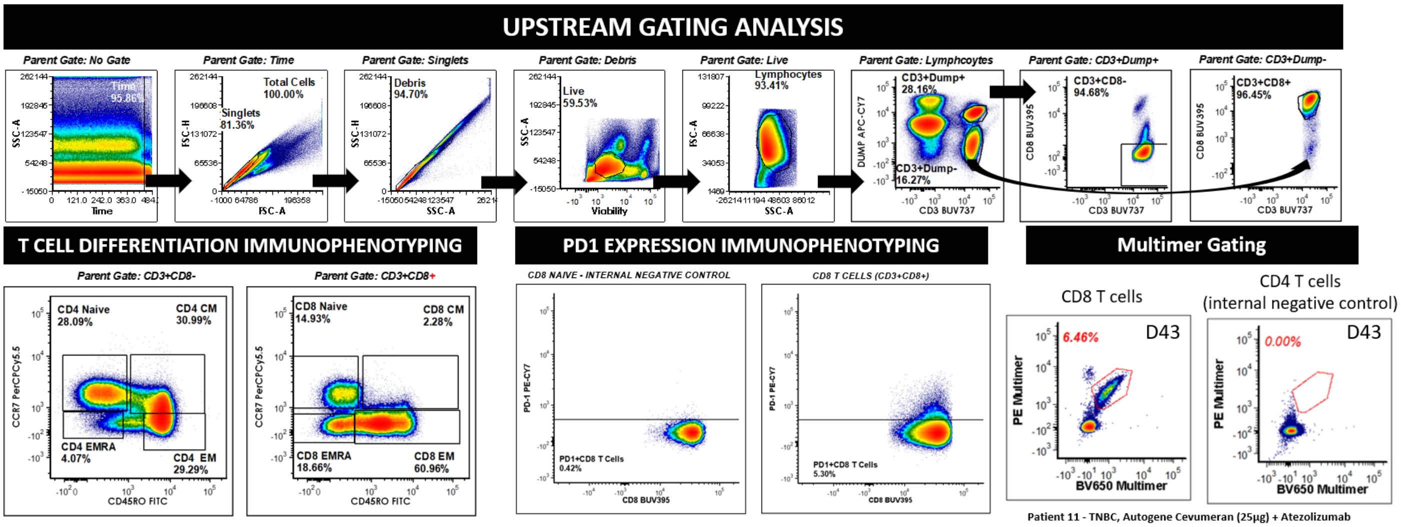

Supplement: Supplementary file 1 — Supplementary Tables 1–9 and Fig. 1. [file 41591_2024_3334_MOESM1_ESM.pdf]
