# Supplementary material for: Autogene cevumeran with or without atezolizumab in advanced solid tumors: a phase 1 trial
Source: Nat Med. 2025 Jan 6;31(1):152–64. doi: 10.1038/s41591-024-03334-7 (PMC11750724; doi:10.1038/s41591-024-03334-7)
Supplement: Supplementary file 2 — Reporting Summary [file 41591_2024_3334_MOESM2_ESM.pdf]

nature portfolio

Corresponding author(s): Juanita Lopez, Michael Mancuso

Last updated by author(s): Sep 13, 2024

Reporting Summary

Nature Portfolio wishes to improve the reproducibility of the work that we publish. This form provides structure for consistency and transparency in reporting. For further information on Nature Portfolio policies, see our Editorial Policies and the Editorial Policy Checklist.

Statistics

For all statistical analyses, confirm that the following items are present in the figure legend, table legend, main text, or Methods section.

n/a	Confirmed	
☐	☒	The exact sample size (*n*) for each experimental group/condition, given as a discrete number and unit of measurement
☐	☒	A statement on whether measurements were taken from distinct samples or whether the same sample was measured repeatedly
☐	☒	The statistical test(s) used AND whether they are one- or two-sided<br>*Only common tests should be described solely by name; describe more complex techniques in the Methods section.*
☒	☐	A description of all covariates tested
☒	☐	A description of any assumptions or corrections, such as tests of normality and adjustment for multiple comparisons
☐	☒	A full description of the statistical parameters including central tendency (e.g. means) or other basic estimates (e.g. regression coefficient) AND variation (e.g. standard deviation) or associated estimates of uncertainty (e.g. confidence intervals)
☐	☒	For null hypothesis testing, the test statistic (e.g. *F*, *t*, *r*) with confidence intervals, effect sizes, degrees of freedom and *P* value noted<br>*Give P values as exact values whenever suitable.*
☒	☐	For Bayesian analysis, information on the choice of priors and Markov chain Monte Carlo settings
☒	☐	For hierarchical and complex designs, identification of the appropriate level for tests and full reporting of outcomes
☒	☐	Estimates of effect sizes (e.g. Cohen's *d*, Pearson's *r*), indicating how they were calculated

Our web collection on statistics for biologists contains articles on many of the points above.

Software and code

Policy information about availability of computer code

Data collection	iMedidata RAVE EDC
Data analysis	Clinical safety and activity summaries were performed using SAS v9.4 and R v3.6.3 (R packages used: dplyr v0.8.5, tidyr v1.0.2, ggplot2 v3.3.0, gridExtra v2.3, rtables v0.3.8, gtable v0.3.0).<br><br>Genomics-related data analysis was coordinated by a software pipeline implemented in Python 2.7x. DNA reads were aligned to the reference genome hg19 with bwa (version 0.7.10). Alignment files were converted to BAM format using samtools (version 0.1.19).<br><br>For RNA-seq, RNA reads were aligned to the hg19 transcriptome using sailfish (version 0.7.6). Non-expressed transcripts were filtered. RNA reads were aligned to the hg19 reference genome using STAR (version 2.4.2a).<br><br>HLA binding affinity was predicted with T-cell prediction tools (version 2.13, IEDB). An algorithm in R was used to select up to 20 neoantigen autogene cevumeran targets from the list of prioritized MPS based on HLA I and HLA II binding predictions, transcript expression, variant allele frequency and other criteria.<br><br>ELISpot plate scan and QC was performed using AID ELISPOT 7.0 software (AID Autoimmun Diagnostika). T-cell responses stimulated by peptides were compared to T-cell responses stimulated with cell culture medium only as negative control using an in-house ELISpot data analysis tool (EDA), based on two statistical test (distribution-free resampling) according to Moodie et al. (refer to Methods section in the manuscript for references), to provide sensitivity while maintaining control over false positives.

For manuscripts utilizing custom algorithms or software that are central to the research but not yet described in published literature, software must be made available to editors and reviewers. We strongly encourage code deposition in a community repository (e.g. GitHub). See the Nature Portfolio guidelines for submitting code & software for further information.

Data

Policy information about availability of data

All manuscripts must include a data availability statement. This statement should provide the following information, where applicable:

- Accession codes, unique identifiers, or web links for publicly available datasets
- A description of any restrictions on data availability
- For clinical datasets or third party data, please ensure that the statement adheres to our policy

For eligible studies qualified researchers may request access to individual patient-level clinical data through a data request platform. At the time of writing this request platform is Vivli: https://vivli.org/ourmember/roche/. As this study is ongoing, access to patient-level data from this trial will not be available until at least 18 months after the last patient visit and a clinical study report has been completed. After that time, requests for data will be assessed by an independent review panel, which decides whether or not the data will be provided. On average it takes a few months to access data in the Vivli platform, but the timeline will vary depending on the number of data contributors, the number of studies, and your availability to respond to comments. Once approved, the data are available for up to 24 months.
For up to date details on Roche's Global Policy on the Sharing of Clinical Information and how to request access to related clinical study documents, see here: https://www.roche.com/innovation/process/clinical-trials/data-sharing. Anonymized records for individual patients across more than one data source external to Roche can not, and should not, be linked due to a potential increase in risk of patient re-identification.

DNA reads were aligned to the reference genome hg19 with bwa (version 0.7.10).

Research involving human participants, their data, or biological material

Policy information about studies with human participants or human data. See also policy information about sex, gender (identity/presentation), and sexual orientation and race, ethnicity and racism.

Reporting on sex and gender	Sex is reported per protocol
Reporting on race, ethnicity, or other socially relevant groupings	Race was reported in Table 1 and defined per protocol. Included analyses were not based on race, ethnicity, or other socially relevant groupings.
Population characteristics	Population characteristics are reported in Table 1
Recruitment	Patients were recruited from clinics at participating institutions from 33 sites in North America and Europe. These sites were selected based on factors such as patient population availability and expertise. Patients were recruited, screened, and enrolled at the discretion of the investigator. Attempts to limit bias through study design and site selection were made to create a representative population of patients.<br><br>Eligible patients had locally advanced, recurrent or metastatic incurable malignancy that had progressed after at least one available standard therapy; for whom standard therapy does not exist, has proven to be ineffective or intolerable or is considered inappropriate; for whom a clinical trial of an investigational agent is a recognized standard of care; or for whom a clinical trial of an investigational agent in combination with an anti-PD-L1 antibody is considered an acceptable treatment option (phase 1b only). Patients were ≥18 years old with an Eastern Cooperative Oncology Group performance status 0–1 and were required to have ≥5 identified neoantigens to be eligible.
Ethics oversight	The study was conducted in accordance with the Declaration of Helsinki, International Conference on Harmonization E6 guidelines, and Good Clinical Practice guidelines. Approval from institutional review boards and ethics committees was obtained before study start.<br><br>The protocol was approved by the institutional review boards at Karolinska Hospital, UZ Gent, Southampton General Hospital, Stanford Cancer Center, Universitatsmedizin der Johannes Gutenberg Universitat Mainz, Universitaetsklinikum Essen, Massachusetts General Hospital, Sarah Cannon Research Institute- Tennessee Oncology, Hospital Univ Vall d'Hebron Servicio de Oncologia, Columbia University Medical Center, Sint Augustinus Wilrijk, Nationales Centrum fur Tumorerkrankungen Heidelberg, Dana-Farber Cancer Institute, Seattle Cancer Care Alliance, CHU Sart Tilman, Akademiska sjukhuset, Onkologkliniken, UCSF Comprehensive Cancer Center, Antoni van Leeuwenhoek Ziekenhuis, Providence Oncology and Hematology Care Eastside, Fachklinik fur Lungenerkrankungen, The Ottawa Hospital Cancer Centre, Universitair Medisch Centrum Utrecht, University of Oklahoma Health Sciences Center, Clinica Universitaria de Navarra, LungenClinic Groshansdorf, Georgetown University, Beth Israel Deaconess Medical Center, Leiden University Medical Center, University of Pittsburgh Medical Center, and Klinisch-Pharmakologisches Studienzentrum.

Note that full information on the approval of the study protocol must also be provided in the manuscript.

Field-specific reporting

Please select the one below that is the best fit for your research. If you are not sure, read the appropriate sections before making your selection.

☒ Life sciences ☐ Behavioural & social sciences ☐ Ecological, evolutionary & environmental sciences

Life sciences study design

All studies must disclose on these points even when the disclosure is negative.

Sample size	This study planned to enroll 307-770 patients, depending on the number and size of the backfill, dose exploration, dose expansion cohorts, and biomarker and adjuvant substudies. This study intended to obtain preliminary safety, pharmacodynamic, and activity information and the sample sizes do not reflect explicit power and type 1 error considerations.
Data exclusions	All patients who received ≥1 dose of autogene cevumeran or atezolizumab were included in the safety and activity analyses. Data from patients enrolled into an adjuvant NSCLC substudy or into cohorts evaluating prophylactic corticosteroids with autogene cevumeran administration and crossover cohorts are not included in this manuscript.
Replication	Replication is not applicable to this Phase 1 clinical trial evaluating a individualized mRNA Neoantigen-specific immunotherapy in human patients.
Randomization	This was a non-randomized Phase 1a/b study where patients were enrolled directly into autogene cevumeran monotherapy or in combination with atezolizumab treatment arm.
Blinding	This was an open-label study

Reporting for specific materials, systems and methods

We require information from authors about some types of materials, experimental systems and methods used in many studies. Here, indicate whether each material, system or method listed is relevant to your study. If you are not sure if a list item applies to your research, read the appropriate section before selecting a response.

Materials & experimental systems

n/a	Involved in the study	
☐	☒	Antibodies
☒	☐	Eukaryotic cell lines
☒	☐	Palaeontology and archaeology
☒	☐	Animals and other organisms
☐	☒	Clinical data
☒	☐	Dual use research of concern
☒	☐	Plants

Methods

n/a	Involved in the study	
☒	☐	ChIP-seq
☐	☒	Flow cytometry
☒	☐	MRI-based neuroimaging

Antibodies

Antibodies used

CD3, BUV737, BD Biosciences, Clone UCHT1, Catalog No. 612751
CD4, APC-CY7, Biolegend, Clone RPA-T4, Catalog No. 300518
CD14, APC-CY7, BD Biosciences, Clone MϕP9, Catalog No. 557831
CD16, APC-CY7, Biolegend, Clone 3G8, Catalog No. 302018
CD19, APC-CY7, Biolegend, Clone SJ25C1, Catalog No. 363010
CD8, BUV395, BD Biosciences, Clone RPA-T8, Catalog No. 563795
PD-1, PE-CY7, Biolegend, Clone EH12.2H7, Catalog No. 329918
CCR7, PerCPCy5.5, Biolegend, Clone G043H7, Catalog No. 353220
CD45RO, FITC, BD Biosciences, Clone UCHL1, Catalog No. 555492
Live/Dead Fixable Dead Cell Staining Kit, UV excitation, ThermoFisher Scientific, NA, Catalog No. L23105)

Dilution was per vendors' recommendation.

Validation

Commercial antibodies/reagents are validated by the manufacturer. Detailed validation information can be found on the manufacturers' websites, specifically on BioLegend's product pages and BD Biosciences' datasheets.
Biolegend: https://www.biolegend.com/reproducibility
BD Biosciences: https://www.bdbiosciences.com
ThermoFisher Scientific: https://www.thermofisher.com/order/catalog/product/L23105

Clinical data

Policy information about clinical studies

All manuscripts should comply with the ICMJE guidelines for publication of clinical research and a completed CONSORT checklist must be included with all submissions.

Clinical trial registration: ClinicalTrials.gov, NCT03289962

Study protocol: The protocol is included in the Supplementary Information

Data collection: A total of 213 patients were enrolled across 33 sites in North America and Europe between 21 December 2017. Health care professionals at the clinical trial sites collected samples and recorded data into EDC systems.

Outcomes: The primary objective was safety and tolerability of autogene cevumeran as monotherapy and in combination with atezolizumab. Safety was assessed through summaries of DLTs, AEs, changes in laboratory test results, changes in vital signs and ECGs and exposure to study treatment. AEs were measured per NCI CTCAE, version 5.0.

Exploratory objectives included the characterization of the pharmacokinetic and pharmacodynamic profile of autogene cevumeran as well as the preliminary anti-tumor activity and immunogenicity.

Pharmacodynamic biomarkers were assessed from patients with adequate tumor tissue and/or blood for analysis. Immune response induced by autogene cevumeran was assessed by ex vivo IFNγ-ELISpot in all patients who had reached cycle 4 and from whom sufficient material was available. Response was assessed by the investigator based on physical examinations and imaging modalities using RECIST v1.1, Response Evaluation Criteria in Solid Tumours version 1.1 (RECIST v1.1). The sponsor (Genentech) derived the BOR and confirmed BOR per RECIST v1.1 based on entries for all target lesions, nontarget lesions and new lesions up to the clinical cut-off date.

Plants

Seed stocks: N/A

Novel plant genotypes: N/A

Authentication: N/A

Flow Cytometry

Plots

Confirm that:

- [x] The axis labels state the marker and fluorochrome used (e.g. CD4-FITC).
- [x] The axis scales are clearly visible. Include numbers along axes only for bottom left plot of group (a 'group' is an analysis of identical markers).
- [x] All plots are contour plots with outliers or pseudocolor plots.
- [x] A numerical value for number of cells or percentage (with statistics) is provided.

Methodology

Sample preparation: Cryopreserved PBMCs from clinical trial subjects were thawed, stained and washed for multimer staining and immunophenotyping

Instrument: BD LSR Fortessa

Software: Software to collect: DIVA
Software to analyze flow cytometry data: FCS Express

Cell population abundance: No cell sorting was performed

Gating strategy: The process begins with the utilization of SSC-A/Time to ensure proper acquisition and lead to the creation of the Time gate.

Gating strategy

Subsequently, cells within the "Time" gate undergo analysis based on FSC-H/FSC-A to perform doublet clean-up, resulting in a Singlets gate. Cells within this gate are further examined using SSC-H/SSC-A to eliminate any debris. The gated cells are then visualized on SSC-A/Viability to establish a gate for viable cells. The viable cells are subjected to analysis through FSC-A/SSC-A to create a gate for Lymphocytes.
From the lymphocyte gate, the "dump channel" adjacent to CD3 is employed to extract CD14+, CD16+, and CD19+. Using the same plot, CD3+Dump- and CD3+Dump+ gates are generated. Subsequently, these cells are plotted on CD8 alongside CD3, resulting in CD3+CD8+ T Cells and CD3+CD8-(CD4+) T Cells, respectively.
T Cell subpopulations are further gated by analyzing CCR7/CD45RO expression, leading to the identification of Naïve, Central Memory (CM), Effector Memory (EM), and Terminally Differentiated Effector Memory (EMRA). Also, PD1 expression was analyzed on CD3+CD8+ T Cells; CD8 Naïve cells guided gate placement, behaving as internal negative control.
Multimer analysis was done on CD3+CD8+ in order to identify any antigen-specific multimer positive CD8 T cells; multimer analysis on CD3+CD8-(CD4+) T Cells were used as internal negative control. Then, multimer positive CD3+CD8+ T Cells underwent immunophenotyping analysis, similarly to CD3+CD8+ T Cells, to identify their T cell differentiation status and PD1 expression levels.
Gates are established on fluorescence plots to differentiate these populations. This differentiation is achieved by separating cells exhibiting low fluorescence (indicative of negative populations) from those with high fluorescence (indicative of positive populations).

☒ Tick this box to confirm that a figure exemplifying the gating strategy is provided in the Supplementary Information.
